# Integrated biosynthesized silver nanoparticles, chitosan, and *Trichoderma asperelloides* for managing onion white rot under greenhouse conditions

**DOI:** 10.1038/s41598-026-56842-6

**Published:** 2026-06-16

**Authors:** Samah A. El-Debaiky, Saida M. Amer, Aya A. El-Shafay, Mohamed Abou-Zeid, Yehia A.-G. Mahmoud

**Affiliations:** 1 Mycology Research Lab, Botany and Microbiology Department, Faculty of Science, Tanta University, Tanta, 31527 Egypt; 2 Wheat Diseases Research Department, Plant Pathology Research Institute, Agricultural Research Center, Giza, 12619 Egypt

**Keywords:** Onion white rot, *Sclerotium cepivorum*, *Trichoderma asperelloides*, Silver nanoparticles, Chitosan, Greenhouse study, Biological control, Disease management, Oxidative stress, Biochemistry, Biological techniques, Biotechnology, Microbiology, Nanoscience and technology, Plant sciences

## Abstract

Onion white rot, caused by *Sclerotium cepivorum* (*Stromatinia cepivora*), remains difficult to manage because of the long-term persistence of sclerotia in soil and the limited efficacy of conventional control methods. In this greenhouse study, we evaluated an integrated eco-friendly strategy comprising *Trichoderma asperelloides*, biologically synthesized silver nanoparticles (AgNPs), and an AgNP–chitosan formulation to suppress onion white rot and improve plant performance. The pathogen and antagonistic isolate were identified morphologically and molecularly, and the synthesized nanomaterials were physicochemically characterized. Among all treatments, the combined AgNP–chitosan + *T. asperelloides* treatment showed the strongest effect, reducing disease severity by 94.3% relative to the infected control and significantly improving bulb fresh weight, leaf length, and leaf number. This treatment also reduced malondialdehyde and hydrogen peroxide contents and was associated with increased antioxidant enzyme activities. These findings suggest that the integrated treatment may act through a synergistic combination of direct pathogen suppression and improved regulation of oxidative stress in treated plants. Overall, the results support the potential of this nano-biological strategy for managing onion white rot under controlled greenhouse conditions, although field validation is still required.

## Introduction

Onion (*Allium cepa* L.) is one of the most economically important vegetable crops worldwide and is widely cultivated for both local consumption and export. However, its productivity is severely constrained by several soil-borne diseases, among which white rot is one of the most destructive.

The fungus *Sclerotium cepivorum* (*Stromatinia cepivora*) Berk is a highly specialized, soil-borne pathogen that causes white rot disease, especially in onions (*Allium cepa* L.). The pathogen is considered one of the most damaging diseases affecting Allium crops globally, causing significant yield reductions and persistent soil infestation^1,2^. According to Abd El-Rahim and Amein^3^, white rot primarily occurs under cool (10–20 °C) and moist soil conditions that promote the germination of the pathogen’s survival structures, called sclerotia. Infected plants show signs of foliar chlorosis, premature leaf senescence, and wilting, which are accompanied by extensive decay of the roots and basal plate. The bulb base is usually covered with a white, cottony mycelium that contains many tiny, black sclerotia measuring 100–300 µm in diameter^4^. In the absence of a host, these sclerotia enable *S. cepivorum* to survive in soil for more than 10 years^5^. Germination of sclerotia is triggered by volatile sulfur compounds, such as diallyl disulfide and allyl sulfides, released from Allium root exudates^6,7^. This host-specific chemical signaling explains the restricted host range and epidemiological persistence of *S. cepivorum* in Allium-growing regions. Following germination, the pathogen rapidly colonizes host tissues by secreting pectinolytic and cellulolytic enzymes, leading to cell wall degradation and tissue maceration^3^**.**

The long-term survival of sclerotia and the low efficacy of commercial fungicides make managing white rot challenging. Crop rotation, chemical control, and soil disinfestation have yielded mixed results, and integrated management techniques are often needed^8^.

Sammour et al.^9^ reported that the addition of Eucalyptus leaves and fruit extracts was associated with reduced incidence of onion white rot. Also, the use of household bleach (sodium hypochlorite), sulfur powder, and/or calcium oxide in field experiments proved more effective against *S. cepivorum* infection in onion. Moreover, the use of volatile sulfur compounds to cause sclerotia to germinate is suicidal^10,11^. To lessen the effects of the illness, recent research has focused on developing resistant cultivars and biological control agents^12^**.** Mahmoud et al.^13^ have concluded that the dark pigment contained in *S. cepivorum* sclerotia might play an important role in the aggressiveness of the *S. cepivorum* strain. Melanin may contribute to the pathogen’s virulence and prolonged survival in soil. *S. cepivorum* remains a significant obstacle to sustainable onion production in spite of these efforts. Chitosan, a natural biopolymer derived from chitin, has gained significant attention as an effective antifungal agent, as evidenced by numerous studies demonstrating its efficacy against various phytopathogenic fungi. Its antifungal activity is attributed to its ability to disrupt fungal cell membranes, inhibit spore germination, and induce plant defense responses. Hence, loading silver nanoparticles (AgNPs) onto chitosan is a promising approach that combines AgNPs’ strong antimicrobial properties with chitosan’s biocompatibility and film-forming capability^14,15^**.**

Despite previous studies on biological control agents, chitosan-based treatments, and silver nanoparticles, limited information is available on the integrated use of biosynthesized AgNPs, chitosan, and *Trichoderma asperelloides* for the management of onion white rot. Based on the persistence of *S. cepivorum* sclerotia in soil and the limitations of conventional control strategies, we hypothesized that combining biosynthesized AgNPs, chitosan, and *T. asperelloides* would provide complementary antifungal and plant-protective effects. The novelty of this work lies in evaluating the integrated AgNP chitosan *T. Asperelloides strategy against onion white rot under greenhouse conditions, while linking disease suppression with plant growth, oxidative stress markers, and antioxidant defense* responses. Therefore, this study aimed to evaluate the individual and combined effects of these treatments on onion white rot severity, plant growth, oxidative stress markers, and antioxidant defense responses under greenhouse conditions.

## Material and methods

### Pathogen isolation

*S. cepivorum*, responsible for onion white rot, was recovered from symptomatic onion plants collected across several locations within El-Gharbia Governorate. Sclerotia were excised from infected bulbs and thoroughly rinsed under running tap water, then surface-disinfected in 0.5% sodium hypochlorite for 2 min, followed by three rinses with sterile distilled water. After blotting the material dry between two sterile layers of filter paper to remove residual moisture, the sclerotia were placed on potato dextrose agar (PDA) for fungal growth. Plates were incubated at 18–20 °C for 5–7 days, and emerging colonies were subsequently purified using the hyphal-tip technique to ensure uniformity of the culture. The resulting isolates were maintained on PDA slants at 4 °C for later experiments^16^. A total of three *S. cepivorum* isolates were recovered from infected onion plants. These isolates were preliminarily assessed based on their cultural and pathogenic characteristics, including stability of colony morphology, vigorous mycelial growth, consistent sclerotial development on PDA medium, and ability to produce typical onion white rot symptoms during preliminary assessment. The isolate selected for subsequent greenhouse experiments showed the most stable and consistent characteristics across these observations. No dedicated molecular or biochemical analysis of virulence factors was performed in the present study.

#### Identification of the pathogen

Conventional morphological characteristics were used to characterize the isolated and purified pathogen. Macroscopic identification involved examining colony features, including color, texture, and growth rate. Colony pigmentation, aerial mycelia, and sporulation patterns were also recorded using taxonomic keys^17^.

Cultures were sent to the Molecular Biology Research Unit, Assiut University, for genomic DNA extraction using the Pathogen-Gene Spin DNA/RNA extraction kit (Intron Biotechnology, Korea). Molecular assays were then performed to confirm the identity of the fungal isolate. Purified DNA was subsequently forwarded to SolGent (Daejeon, South Korea) for PCR amplification and sequencing of the rRNA internal transcribed spacer (ITS) region. PCR reactions were prepared using the ITS1 (forward) and ITS4 (reverse) primers, with the primer sequences ITS1 (5′-TCC GTA GGT GAA CCT GCG G-3′) and ITS4 (5′-TCC TCC GCT TAT TGA TAT GC-3′). For sequencing of the amplified product, dNTPs and the same primers were included in the sequencing reaction mixture^18^.

The resulting sequences were edited and aligned, then queried against the National Center for Biotechnology Information (NCBI) database using the Basic Local Alignment Search Tool (BLAST) to determine similarity to closely related taxa. Based on the ITS rRNA gene dataset, a neighbor-joining phylogenetic tree was constructed in MEGA X to support molecular identification and infer relatedness among reference sequences^19^.

### Isolation and identification of antagonistic fungi (Trichoderma spp.)

Soil samples were collected from different localities of Kafar El-Zayat city, EL-Gharbia Governorate, Egypt, from banana, tomato, and onion cultivated soil to isolate *Trichoderma* *spp*. Samples were brought to the laboratory and stored at 4 °C until used. Five-fold serial dilutions of each soil sample were prepared in sterile distilled water (10^−1^–10^−5^) by suspending 10 g of soil in 90 mL sterile distilled water (10^−1^ dilution), then further diluting to 10^−5^. 0.1 mL from each dilution was poured onto the surface of a PDA plate; this was repeated 3 times**.** Plates were incubated at 28 ± 2 °C for 3–7 days. Morphologically different colonies appearing on the plates were sub-cultured using (PDA) media until pure cultures with the morphology of *Trichoderma* *spp* were obtained (HiMedia, India). The obtained pure cultures were kept at 4 °C on PDA slants for further studies^20^. A total of five *Trichoderma*-like isolates were recovered and preliminarily screened. Based on their antagonistic performance against *S. cepivorum* in the dual-culture assay, *T. asperelloides* isolate 102 showed the strongest inhibitory activity and was therefore selected for nanoparticle biosynthesis and greenhouse evaluation.

The purified fungal isolate was initially identified based on detailed morphological characteristics observed under both macro- and microscopic examination. Macroscopic identification involved examining colony features such as color, texture, and growth rate on Potato Dextrose Agar (PDA) medium, incubated at 25 ± 2 °C for 5–7 days. Colony pigmentation, mycelial, and sporulation patterns were also recorded. Microscopically, fungal structures were stained with lactophenol-cotton blue and examined under a compound light microscope. Key features assessed included hyphal morphology, septation, branching patterns, conidial shape, size, arrangement, and spore-bearing structures such as conidiophores or sporangia. These observations were compared with taxonomic keys and standard mycological references for fungal genera and species-level identification. To overcome the limitations of morphological methods, especially in differentiating closely related species or cryptic taxa, molecular approaches targeting ITS-rDNA sequencing were also employed for confirmation, as mentioned above, and species-level resolution^17^.

Molecular identification based on ITS sequence analysis relies on comparing sequences from the recovered isolates with curated database entries to quantify similarity and support taxonomic assignment. As a quality check, the molecular outcome should be consistent with phenotypic characterization rather than contradict it. Accordingly, ITS (internal transcribed spacer) sequences were aligned and queried against GenBank records using the Basic Local Alignment Search Tool (BLAST), enabling the retrieval of the closest-matching fungal ITS sequences. Using this approach, the pathogen was clearly resolved, showing a sequencing identity of > 99%.

Following initial identification, the sequences were trimmed and edited, and a sequence matrix was generated; the finalized dataset was saved in fas format to support phylogenetic reconstruction. For confirmatory work, antagonistic fungal cultures were submitted to the Molecular Biology Research Unit at Assiut University for DNA extraction using the Pathogen-Spin DNA/RNA extraction kit (Intron Biotechnology, Korea). Extracted fungal DNA was then sent to SolGent (Daejeon, South Korea) for PCR amplification and rRNA gene sequencing. PCR was conducted using ITS1 (forward) and ITS4 (reverse) primers added to the reaction mixture, with primer sequences ITS1 (5′-TCC GTA GGT GAA CCT GCG G-3′) and ITS4 (5′-TCC TCC GCT TAT TGA TAT GC-3′); sequencing of the purified PCR product was performed using dNTPs and the same primers^18^. The resulting sequences were analyzed using the NCBI BLAST platform and compared with deposited sequences in the NCBI database to determine homology with the most closely related species. Finally, MEGA X was used to construct a neighbor-joining phylogenetic tree based on the ITS rRNA gene to further substantiate identification^19^.

### Dual culture technique

The antagonistic potential of the isolated *Trichoderma* sp. against *S. cepivorum* was evaluated using a dual-culture assay. Briefly, 5-mm agar plugs were excised from 7-day-old cultures of the *Trichoderma* isolate and *S. cepivorum*, then positioned on opposite sides of Petri dishes containing 15 mL potato dextrose agar (PDA). Plates inoculated with *S. cepivorum* alone served as the control treatment, and each assay was performed in three replicates. All plates were incubated at 18–20 °C and monitored until the pathogen in the control plates achieved full colony coverage.

Antagonism was then quantified using the five-class scale described by Bell et al.^21^. In Class 1, the antagonist entirely covered the medium and completely overgrew the pathogen; in Class 2, the antagonist overgrew at least two-thirds of the plate surface. Class 3 represented approximate equilibrium, where both organisms colonized about half of the medium surface (i.e., more than one-third and less than two-thirds) without clear dominance. In Class 4, the pathogen colonized at least two-thirds of the medium and appeared to resist invasion by the antagonist, whereas in Class 5, the pathogen fully occupied the plate and completely overgrew the antagonist. Among the five *Trichoderma*-like isolates screened, *T. asperelloides* isolate 102 showed the strongest antagonistic activity against *S. cepivorum*. The isolate was classified as Bell scale class 2, as it overgrew approximately two-thirds of the plate surface and markedly restricted pathogen growth. Therefore, isolate 102 was selected for nanoparticle biosynthesis and greenhouse evaluation^22,23^.

### Biosynthesis (Green synthesis) of silver nanoparticles from *Trichoderma* sp.

Silver nanoparticles were synthesized extracellularly using the culture filtrate of the isolated *Trichoderma* sp. In brief, 50 mL of 1 mM aqueous silver nitrate (AgNO₃) was combined with an equal volume (50 mL) of *Trichoderma* sp. culture filtrate in a 250 mL conical flask, after which the pH was adjusted to 8.5. The resulting mixture was incubated at 40 °C under shaking (200 rpm) in the dark for 5 days to facilitate bioreduction. Parallel uninoculated controls were prepared and processed under identical conditions to verify that Ag^+^ reduction was attributable to biological activity rather than abiotic factors. Under these controlled physicochemical parameters, the procedure yielded effective biosynthesis of silver nanoparticles^24^.

### Characterization of silver nanoparticles

#### UV–visible spectroscopy

The reduction of Ag + ions by the culture filtrate of *Trichoderma* sp. and the formation of silver nanoparticles were characterized by UV–visible spectroscopy, with aqueous samples (2 mL) collected and spectra measured. The UV–Vis spectra of these samples were measured on a Cecil model 9200 Ultraviolet visible spectrophotometer operated at a resolution of 1 nm^25^.

#### Transmission electron microscopy (TEM)

Silver nanoparticles produced using the *Trichoderma* sp. culture filtrate were characterized by transmission electron microscopy (TEM; JEOL 100SX, Japan, equipped with an AMT digital camera). To minimize aggregation and enable visualization of discrete particles, samples were ultrasonically dispersed prior to analysis. Subsequently, one to two drops of the resulting suspension were placed onto holey carbon–coated copper grids and dried under an infrared lamp. After 72 h of incubation, the nanoparticle films on the grids were examined and imaged.

TEM provided high-resolution micrographs that allowed direct assessment of nanoparticle morphology, including particle shape and size distribution. This analysis offered detailed structural information essential for interpreting the formation characteristics of the biosynthesized silver nanoparticles^26^.

#### Scanning electron microscope (SEM) and (EDX) analysis

The surface morphology of the biosynthesized silver nanoparticles was examined using scanning electron microscopy (SEM). For sample preparation, a small amount of the dried nanoparticle powder was mounted on a carbon-coated copper grid, and SEM micrographs were recorded to document particle morphology and surface features^27^.

#### (EDX) analysis

Energy Dispersive X-ray Spectroscopy (EDX). This technique is commonly used in conjunction with scanning electron microscopy (SEM) to provide elemental composition information alongside morphological imaging. It works by directing a high-energy electron beam onto the sample surface, exciting atoms and causing characteristic X-rays unique to each element to be emitted. These emitted X-rays are detected and their energies measured, allowing qualitative and quantitative identification of the elements present in the sample^28^.

#### X-ray diffraction (XRD)

Silver nanoparticle formation was further examined by X-ray diffraction (XRD) using an X-ray diffractometer (Phillips PW 1729/40 generator and diffractometer, one-line reactor) equipped with Cu Kα radiation (λ = 1.5405 Å). Diffraction patterns were recorded over a broad range of Bragg angles (2θ = 20–80). For analysis, thin films of the silver nanoparticles were prepared on glass slides; to facilitate efficient sample loading, the slides were first coated with silica gel and then loaded with the silver nanoparticle solution^29^.

#### The Fourier transform infrared spectroscopy (FTIR) spectrum

Fourier-transform infrared (FTIR) spectra were acquired using a Perkin-Elmer 1430 FTIR spectrophotometer. For sample preparation, 5 mg of the material was thoroughly blended with 200 mg of FTIR-grade KBr and pressed into a pellet. The pellet was then mounted in the sample holder, and spectra were recorded across the range of 400–4000 cm^−1^^30^.

#### Zeta potential analysis

Zeta potential, hydrodynamic particle size, and size distribution were determined by dynamic light scattering (DLS) using a Zetasizer Nano ZN at a fixed scattering angle of 173°. Measurements were conducted at 25 ± 0.1 °C to maintain consistent thermal conditions and support a reliable assessment of the colloidal stability of the synthesized silver nanoparticles^31^.

### Preparation of chitosan-loaded silver nanoparticles

Chitosan solution was prepared by dissolving 2 g of chitosan powder (100–300 kDa; Nanotech Company, 6 October City, Cairo, Egypt) in 100 mL of 3% (v/v) glacial acetic acid, with continuous mixing until fully dispersed. Subsequently, 1 mL of glycerin was added under stirring, and the mixture was further agitated to ensure complete dissolution. The solution was then left to stand until it became fully transparent, yielding the chitosan material.

To formulate the AgNPs/chitosan (1:1) composite, 20 mL of the chitosan solution was mixed with 20 mL of the prepared AgNPs suspension, and the mixture was stirred for 30 min to promote uniform blending^32^.

### Characterization of chitosan-loaded silver nanoparticles

The loading of silver nanoparticles biosynthesized by the *Trichoderma* sp. culture filtrate onto chitosan, as well as the formation of the chitosan–silver nanoparticle composite, was confirmed using the same characterization approaches described above for AgNPs. Specifically, UV–visible spectroscopy, transmission electron microscopy (TEM), scanning electron microscopy (SEM), X-ray diffraction (XRD), Fourier-transform infrared (FTIR) spectroscopy, and zeta potential analysis were used to verify nanoparticle incorporation and assess the physicochemical properties of the resulting chitosan-based nanomaterial.

## Greenhouse experiment

### Inoculum preparation

Glass flasks with a capacity of 500 mL were filled with 100 g of rice husk and supplemented with 50 mL of sterile distilled water. The bottles were sterilized in an autoclave at 121 °C for 20 min to ensure complete sterilization. After cooling, each flask was inoculated with five mycelial discs (5 mm diameter) taken from a 10-day-old culture of *S. cepivorum*. The inoculated flasks were incubated at 18–20 °C for 25 days to allow thorough colonization by the fungus, resulting in a robust fungal inoculum. This inoculum was subsequently mixed with sterilized soil at a rate 2% fungus to soil (w/w)^33^.

### Experimental design

First, the soil was sterilized in an autoclave at 121 °C and 1.5 atm for 30 min. The experiment was conducted in plastic pots (upper diameter approximately 18 cm, lower diameter approximately 14 cm, height 18 cm), each containing 3 kg of soil. The sterilized soil was then inoculated with *S*. *cepivorum* (a mixture of sclerotia and mycelia grown on rice husk) at 2% (w/w), equivalent to 20 g of inoculum per 1 kg of soil. Accordingly, 60 g of *S. cepivorum* inoculum was added to each pot. The infested soil was moistened and incubated for two weeks before transplanting. Healthy 45-day-old onion seedlings (cv. Giza 20) were soaked in each treatment solution for 15 min prior to transplanting and then transplanted into the pots. The experiment consisted of three replicates, with 12 pots per replicate. Each treatment solution was applied at 15-day intervals, with each pot receiving 60 g of *S. cepivorum* inoculum per 3 kg of soil. All treatments were applied as soil drenches, including AgNPs (10 mg/L), chitosan (5%), *T. asperelloides* (1 × 10⁸ cfu/mL), tebuconazole 25% EC (fungicide reference treatment at 446.5 cm^3^/ha), and combinations thereof. Chitosan was evaluated as an individual greenhouse treatment and as part of the AgNP–chitosan formulation; no separate in vitro antifungal assay of chitosan alone was conducted.

The greenhouse treatments evaluated in the present study, including control treatments, pathogen status, treatment concentrations, and seedling pretreatment procedures, are summarized in Table 1 to facilitate experimental reproducibility and treatment comparison.

**Table 1:** Treatment matrix used in the greenhouse experiment for onion cultivar (Giza 20), White rot management.

Treatment code	Treatment description	Treatment concentration/dose	Pathogen status	Seedling pretreatment
HC	Healthy control (untreated)	None	Non-infected	Seedlings soaked in sterile distilled water for 15 min
IC	Infected control (untreated)	60 g *S. cepivorum* prepared inoculum per 3 kg soil	Infected with *S. cepivorum*	Seedlings soaked in sterile distilled water for 15 min
F	Fungicide reference treatment (Tebuconazole)	Tebuconazole 25% EC; 446.5 cm^3^/ha	Infected with *S. cepivorum*	Seedlings soaked in tebuconazole solution for 15 min
CHN	Chitosan treatment	5%	Infected with *S. cepivorum*	Seedlings soaked in chitosan solution for 15 min
AgNPs	Biosynthesized silver nanoparticles	10 mg/L	Infected with *S. cepivorum*	Seedlings soaked in AgNP solution for 15 min
TA	*T. asperelloides* isolate 102	1 × 10^⁸^ cfu/mL	Infected with S. cepivorum	Seedlings soaked in *T. asperelloides* suspension for 15 min
AgNPs–CHN	Biosynthesized silver nanoparticles loaded into chitosan	AgNPs (10 mg/L) + chitosan (5%)	Infected with *S. cepivorum*	Seedlings soaked in AgNPs–chitosan formulation for 15 min
AgNPs–TA	AgNPs combined with *T. asperelloides*	AgNPs 10 mg/L + *T. asperelloides* 1 × 10⁸ cfu/mL	Infected with S. cepivorum	Seedlings soaked in AgNPs–*T. asperelloides* suspension for 15 min
AgNPs–CHN–TA	AgNPs loaded into chitosan combined with *T. asperelloides*	AgNPs 10 mg/L + chitosan 5% + *T. asperelloides* 1 × 10⁸ cfu/mL	Infected with *S. cepivorum*	Seedlings soaked in AgNPs–chitosan *T. asperelloides* formulation for 15 min

Sterilized soil was infested with S. cepivorum inoculum at 2% (w/w), equivalent to 60 g inoculum per pot containing 3 kg soil. The experiment was conducted with three replicates, each with 12 pots. After transplanting, treatments were applied as soil drenches at 15-day intervals.

### Extraction and estimation of photosynthetic pigments

Photosynthetic pigments (chlorophyll a, chlorophyll b, and carotenoids) were quantified in leaf tissues using a spectrophotometric procedure. Briefly, 0.1 g of fresh leaves was homogenized in 5 mL of 85% (v/v) aqueous acetone for 5 min. The homogenate was centrifuged, and the resulting supernatant was brought to a defined final volume with 85% acetone. Absorbance of the extract was read against an 85% acetone blank at 663, 644, and 452.5 nm using a spectrophotometer, following the method recommended by Metzner et al.^34^. Pigment concentrations in the leaf extracts were then calculated (µg/mL) using the following equations:

$${\text{Chl a }} = { 1}0.{\text{3 E663 }}{-} \, 0.{\text{918 E644}}$$

$${\text{Chl b }} = { 19}.{\text{7 E644 }}{-}{ 3}.{\text{87 E663}}$$

$${\text{Carotenoids }} = { 4}.{\text{2 E452}}.{5 } - \, \left( {0.0{264 } \times {\text{ chl a }} + \, 0.{426 } \times {\text{ chl b}}} \right)$$

where E denotes the absorbance, then the pigment content was calculated as mg/g dry weight as follows:

$$\frac{Reading \times Dilution}{{Sample \;volume \times Dry \times wt}} \times 1000$$

### Estimation of total soluble sugars

#### Extraction

The plant materials (root and shoot) were dried in an oven at 50 °C to constant weight, then ground to a fine powder. According to Naguib et al.^35^, dried fine powder material (100 mg) of either root or shoot was added to 5 mL borate buffer (28.63 g boric acid + 29.8 g potassium chloride + 3.5 g sodium hydroxide in 1 L solution, pH 8.0) and incubated overnight, then centrifuged.

Sterilized soil was infested with *S. cepivorum* inoculum at 2% (w/w), equivalent to 60 g inoculum per pot containing 3 kg soil. The experiment was conducted with three replicates, each with 12 pots. After transplanting, treatments were applied as soil drenches at 15-day intervals.

#### Estimation of total soluble sugars

Total soluble sugars were quantified following the procedure of Dubois et al.^36^. Briefly, 0.1 mL of the borate buffer extract was mixed with 1 mL of 5% phenol, after which 5 mL of concentrated sulfuric acid was added. The reaction mixture was then incubated in a water bath at 25 °C for 20 min to allow color development. Absorbance was measured at 490 nm, and total soluble sugar content was calculated from a standard calibration curve and expressed as mg/g d.wt.

### Estimation of hydrogen peroxide (H_2_O_2_) content

Hydrogen peroxide (H₂O₂) content was determined according to the method recommended by Velikova et al.^37^. Briefly, 100 mg of leaf tissue was homogenized in 5 mL of 0.1% trichloroacetic acid (TCA) and centrifuged at 12,000 rpm for 15 min. An aliquot (0.5 mL) of the supernatant was then combined with 0.5 mL of 10 mM potassium phosphate buffer (pH 7.0) and 1 mL of 1 M potassium iodide, and absorbance was measured at 390 nm. H₂O₂ concentration was calculated using an extinction coefficient of 0.28 µM^−1^ cm^−1^ and expressed as µmol/g f.wt.

### Determination of malondialdehyde content (MDA)

Malondialdehyde (MDA) content was quantified following Heath and Packer^38^ using the thiobarbituric acid (TBA) assay, which estimates TBA-reactive substances as an indicator of lipid peroxidation. Briefly, 0.5 g of fresh leaf tissue was homogenized in 10 mL of 5% (w/v) trichloroacetic acid (TCA) and centrifuged at 4000 rpm for 15 min. An aliquot (2 mL) of the supernatant was then combined with 2 mL of 0.677% (w/v) TBA, and the mixture was heated in a boiling water bath for 15 min. After rapid cooling in cold water for 10 min, absorbance was recorded at 532 and 600 nm. MDA concentration (n. mole/g f.wt.) was calculated using an extinction coefficient of 155 µM^−1^ cm^−1^.

### Estimation of ascorbic acid content (ASA)

Ascorbic acid (ASA) content was determined following Oser^39^. Briefly, 0.1 g of fresh leaf tissue was homogenized in 5 mL of 5% (w/v) sulfosalicylic acid and centrifuged at 10,000 rpm for 10 min. For color development, the reaction mixture consisted of 2 mL of 2% sodium molybdate, 2 mL of 0.15 N H₂SO₄, 1 mL of 1.5 mM Na₂HPO₄, and 1 mL of the tissue extract. The mixture was incubated at 60 °C in a water bath for 40 min, then cooled and centrifuged at 3000 rpm for 10 min to clarify the solution. Absorbance was recorded at 660 nm, and ASA concentration was calculated from a standard calibration curve and expressed as mg/g d.wt.

### Estimation of reduced glutathione content (GSH)

Reduced glutathione (GSH) content was quantified as described by Anderson^40^. Briefly, 0.1 g of fresh leaf tissue was homogenized in 5 mL of 3% (w/v) sulfosalicylic acid and centrifuged at 10,000 rpm for 10 min to obtain a clear extract. For the assay, 0.5 mL of the tissue supernatant was mixed with 0.5 mL of 0.5 mM potassium phosphate buffer (pH 7.0) and 50 µL of 3 mM 5,5′-dithio-bis (2-nitrobenzoic acid) (DTNB), with additional reagents added in the required volumes. After 5 min of reaction, absorbance was recorded at 412 nm. GSH concentration was determined from a standard GSH calibration curve and expressed accordingly.

### Estimation of total phenolic content

Total phenolic content was determined following the method of Jindal and Singh^41^. Briefly, 0.1 g of dried tissue was extracted with 10 mL of 95% ethanol, and the resulting clear supernatants were pooled and adjusted to a final volume of 10 mL. An aliquot (1 mL) of the ethanolic extract was combined with 0.1 mL of Folin reagent and 1 mL of 20% Na₂CO₃, then diluted to 5 mL with distilled water. After incubation for 30 min to allow color development, absorbance was recorded at 650 nm. Total phenolics were calculated from a gallic acid standard curve prepared at different concentrations, as described previously, and expressed as mg/g d.wt.

### Estimation of flavonoid compounds

Flavonoid content was quantified using the aluminum chloride colorimetric assay. Briefly, 0.5 mL of the ethanolic plant extract was mixed with 1.5 mL of 95% ethanol, 0.1 mL of 10% aluminum chloride, 0.1 mL of 1 M potassium acetate, and 2.8 mL of distilled water. The mixture was incubated at room temperature for 30 min to allow complex formation, and absorbance was then recorded at 415 nm. For the blank, 10% aluminum chloride was replaced with an equal volume of distilled water. Flavonoid concentration was calculated from a quercetin calibration curve and expressed as mg/g dm^42^.

### Estimation of antioxidant and metabolic enzymes

#### Preparation of enzyme extract

Fresh leaf tissue (1.0 g) was finely ground in a mortar under liquid nitrogen and then homogenized in 10 mL of extraction buffer containing 50 mM potassium phosphate (pH 7.8), 0.5 mM EDTA, 1% polyvinylpyrrolidone (PVP), 1 mM ascorbic acid, and 10% glycerol. The homogenate was clarified by centrifugation at 12,000 rpm for 20 min at 4 °C. The resulting supernatant was collected as the crude enzyme extract and maintained at 4 °C until analysis^43^.

### Antioxidant enzymes

#### Peroxidase (POD)

Peroxidase (POD; EC 1.11.1.7) activity was assayed as described by Kato and Shimizu^44^. The reaction mixture (final volume 3 mL) consisted of 0.1 M sodium phosphate buffer (pH 5.8), 7.2 mM guaiacol, 11.8 mM H₂O₂, and 0.1 mL of enzyme extract. The reaction was started by adding H₂O₂, and the increase in absorbance was monitored at 470 nm. POD activity was calculated using the extinction coefficient of 26.6 mM^−1^ cm^−1^ for tetraguaiacol and expressed as µM/g f.wt min^−1^.

#### Ascorbate peroxidase (APX)

Ascorbate peroxidase (APX; EC 1.11.1.11) activity was determined immediately using freshly prepared enzyme extracts, following the method of Nakano and Asada^45^. The assay was performed in a 1 mL reaction mixture containing 50 mM potassium phosphate buffer (pH 7.0), 0.1 mM hydrogen peroxide, 0.5 mM ascorbate, and 0.1 mM EDTA. Enzyme activity was monitored by tracking the H₂O₂-dependent oxidation of ascorbate as a decrease in absorbance at 290 nm.

#### Glutathione peroxidase (GPX)

Glutathione peroxidase (GPX) activity was assayed according to Hopkins and Tudhope^46^ using *t*-butyl hydroperoxide as the substrate. The reaction mixture contained 50 mM potassium phosphate buffer (pH 7.0), 2 mM EDTA, 0.28 µM NADPH, 0.13 µM glutathione, 0.16 U glutathione reductase, 0.073 µM peroxide, and an appropriate volume of enzyme extract. GPX activity was determined by monitoring the rate of NADPH + H^+^ oxidation as a decrease in absorbance at 340 nm over 6 min, and results were expressed as U mg^−1^ protein.

#### Catalase

Catalase (CAT; EC 1.11.1.6, NC-IUBMB) activity was assayed according to Patterson et al.^47^ by monitoring H₂O₂ decomposition. The reaction mixture (3.0 mL) contained 10.5 mM H₂O₂ prepared in 0.05 M potassium phosphate buffer (pH 7.0). The reaction was initiated by adding 0.1 mL of enzyme extract, and measurements were carried out at 25 °C. CAT activity was calculated from the decline in absorbance at 240 nm using an extinction coefficient (Δε) of 43.6 mM^−1^ cm^−1^ at 240 nm. One unit of CAT activity was defined as the amount of enzyme catalyzing the conversion of 1 mM H₂O₂ min^−1^ at 25 °C.

### Statistical analysis

All experiments were conducted using a completely randomized design. Data obtained for disease severity, growth attributes, and biochemical/enzymatic variables were subjected to analysis of variance (ANOVA) to test for treatment effects. Prior to ANOVA, datasets were examined for compliance with the assumptions of normality and homogeneity of variances; where necessary, percentage data were transformed using the arcsine square-root function to stabilize variances, while non-transformed means are reported for ease of interpretation. When significant differences among treatments were detected, mean values were compared using Duncan’s multiple range test at P < 0.05. Statistical analyses were performed using IBM SPSS Statistics, version 25 (IBM Corp., Armonk, NY, USA). Results are presented as means with their associated variability estimates, and statistical significance was inferred at the 5% level^48^.

## Results

### Isolation of *S. cepivorum*

#### Morphological identification

**Figure 1:**
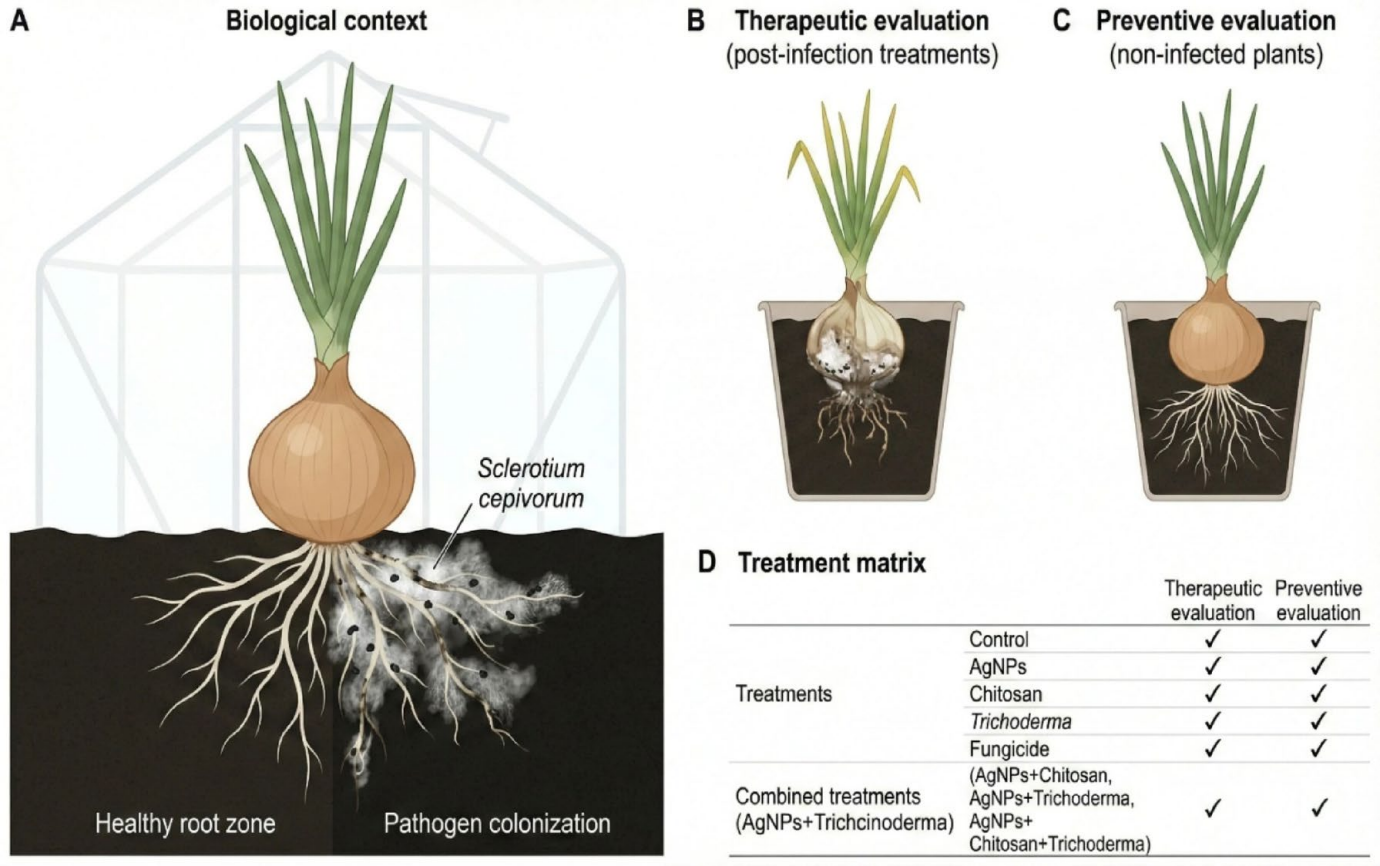
Greenhouse treatment scheme showing the infected and non-infected onion conditions and the treatment matrix used to evaluate AgNPs, chitosan, *T.asperelloides*, tebuconazole, and their combined formulations against *S\. cepivorum*.

The fungal growth on PDA medium appears as dense, white, cottony mycelial colonies that rapidly cover the agar surface. Small, rounded sclerotia initially white to pale gray develop within the mycelial mat and gradually darken with maturation, as shown in (Fig. 1). This morphological pattern, characterized by prolific aerial hyphae and abundant sclerotia production across the culture plate, is typical for the identification and isolation of *S. cepivorum,* the causative agent of white rot disease. Microscopically, *S. cepivorum* is characterized by the presence of filamentous, septated, and multinucleated branched hyphae. The distinctive feature is the production of hardened, compact, and irregularly shaped sclerotia. The sclerotia are initially white to pale, then darken with age as melanin accumulates. These sclerotia serve as survival structures, allowing the fungus to persist in soil under adverse conditions.

#### Molecular identification

Molecular identification of the pathogenic fungal isolate was carried out based on sequencing of the ribosomal DNA region. The obtained sequence was subjected to BLAST analysis against the NCBI GenBank database and showed high similarity to *S. cepivorum* (*Stromatinia cepivora*). Accordingly, the pathogenic isolate was identified as *S. cepivorum* (*Stromatinia cepivora* isolate S. c1), and the sequence was deposited in GenBank under accession number PX089568.1.

### Isolation and identification of *Trichoderma* spp from the soil

The isolated *Trichoderma* spp were examined for identification both morphologically and microscopically.

#### Morphological identification

The isolated *Trichoderma* culture exhibits rapid growth, often filling the plate within 3–5 days. Colonies appear white at first, later turning green due to heavy conidiation; the reverse side is typically colorless to pale yellow to yellow. Microscopic examination shows that hyphae are hyaline, septate, and highly branched. Conidiophores are repeatedly branched, forming a pyramidal or “tree-like” structure. Phialides are flask-shaped, often arranged in whorls, and produce clusters of conidia. Conidia are unicellular, smooth to rough, green, globose to subglobose (2–5 μm) conidia.

#### Molecular identification

**Figure 2:**
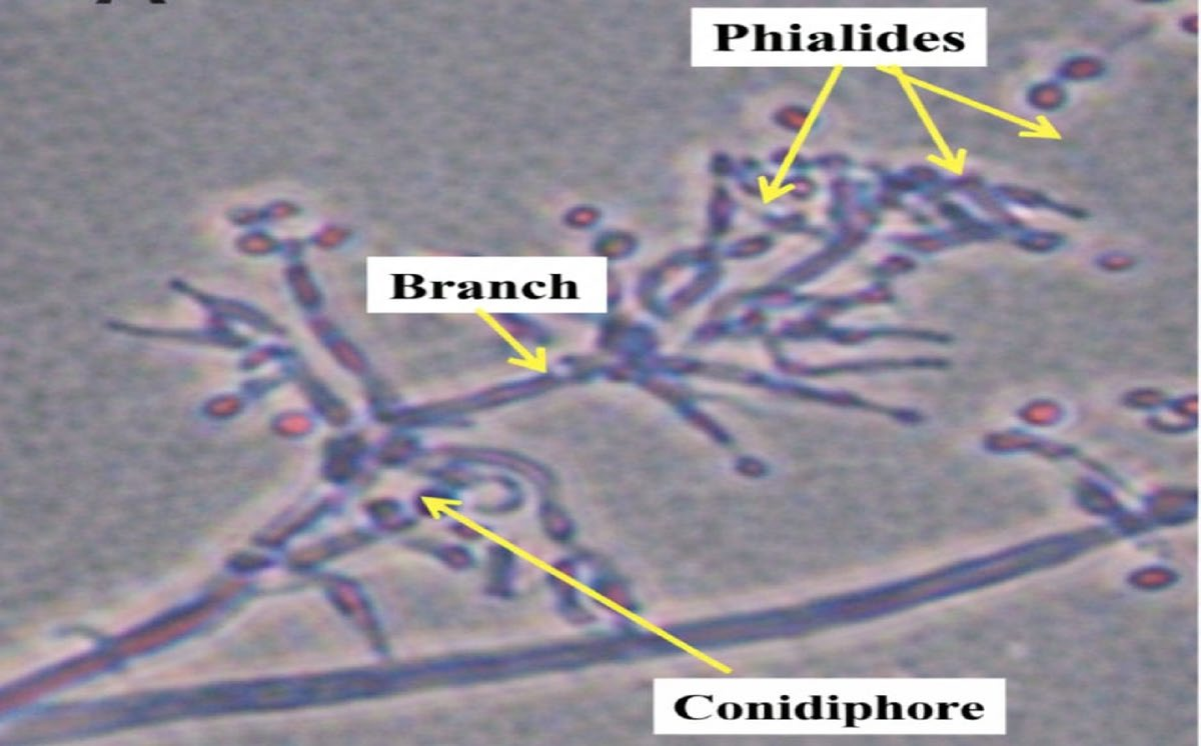
Microscopic examination of *Trichoderma* isolates (A), 1000X.

Molecular identification of the antagonistic fungal isolate was carried out based on sequencing of the ribosomal DNA region. The obtained sequence was subjected to BLAST analysis against the NCBI GenBank database and showed high similarity to *T. asperelloides*. Accordingly, the isolate was identified as *T. asperelloides* isolate 102, and the sequence was deposited in GenBank under accession number PX107871.1 (Fig. 2).

**Figure 3:**
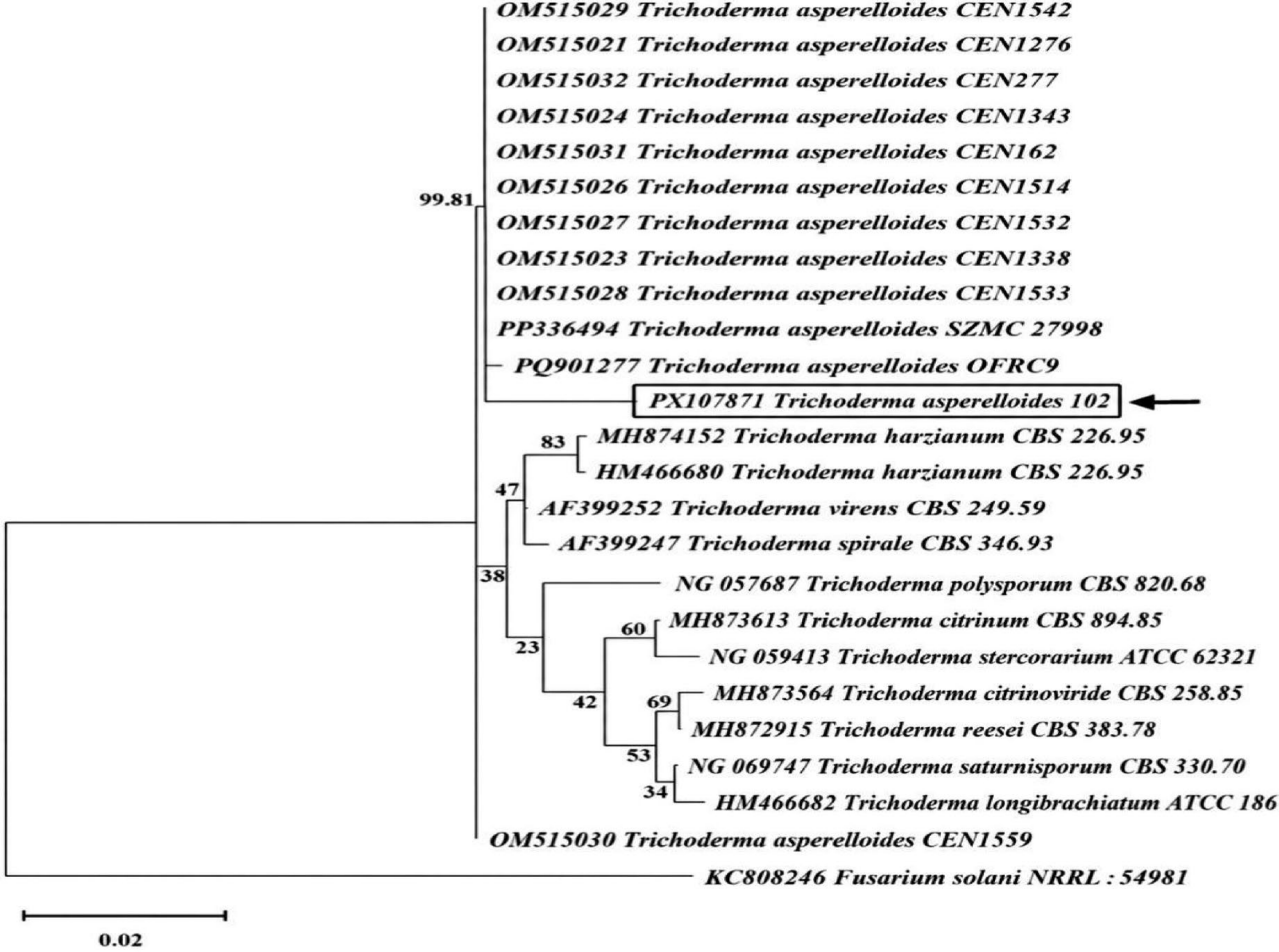
Phylogenetic tree showing the placement of the studied *T. asperelloides* isolate among closely related reference sequences, supporting its molecular identification*.*

The phylogenetic tree in Fig. 3 shows the placement of the studied *Trichoderma asperelloides* isolate (PX107871) among closely related reference sequences. The isolate clustered with reference strains of *T. asperelloides*, including OM515029 and OM515021, supporting its molecular identification. In contrast, more distantly related taxa, such as *T. harzianum* and *T. virens*, were separated from the studied isolate, indicating a clear phylogenetic distinction within the analyzed ITS dataset.

### Dual culture technique

**Figure 4:**
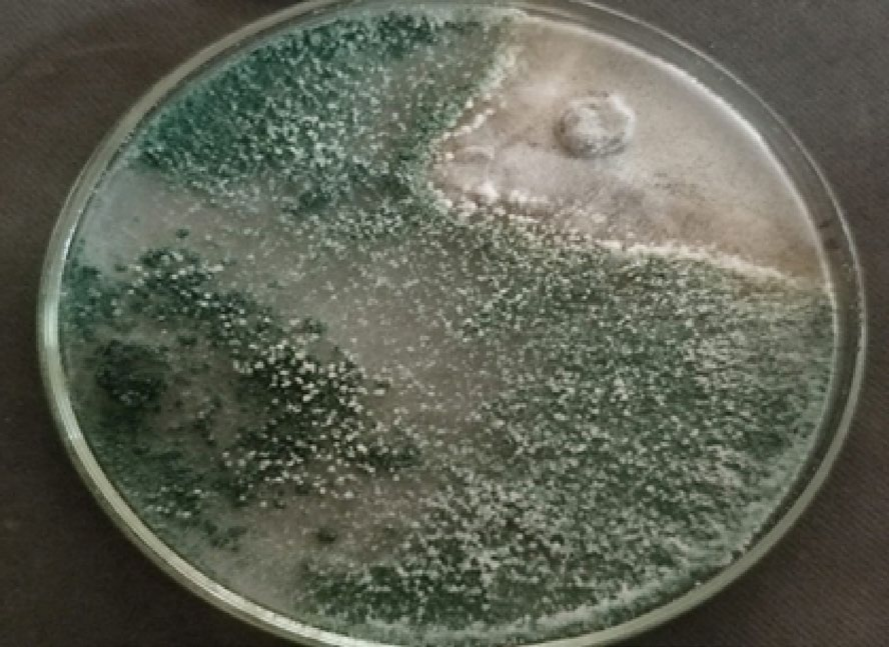
Dual culture assay between *T.asperelloides* and *Sclerotium cepivorum*.

The dual culture assay clearly demonstrates the antagonistic interaction between the tested fungi. The dual-culture plates of the tested *T. asperelloides* and the pathogenic fungus *S. cepivorum* showed clear mycoparasitism of *Trichoderma* spp. over *S. cepivorum* (Fig. 4). The *T. asperelloides* colony showed rapid radial growth, producing abundant green conidia, and progressively overgrew the *S*. *cepivorum* colony. In contrast, the pathogen’s mycelial growth was restricted and suppressed.

### Biosynthesis (Green synthesis) of silver nanoparticles

**Figure 5:**
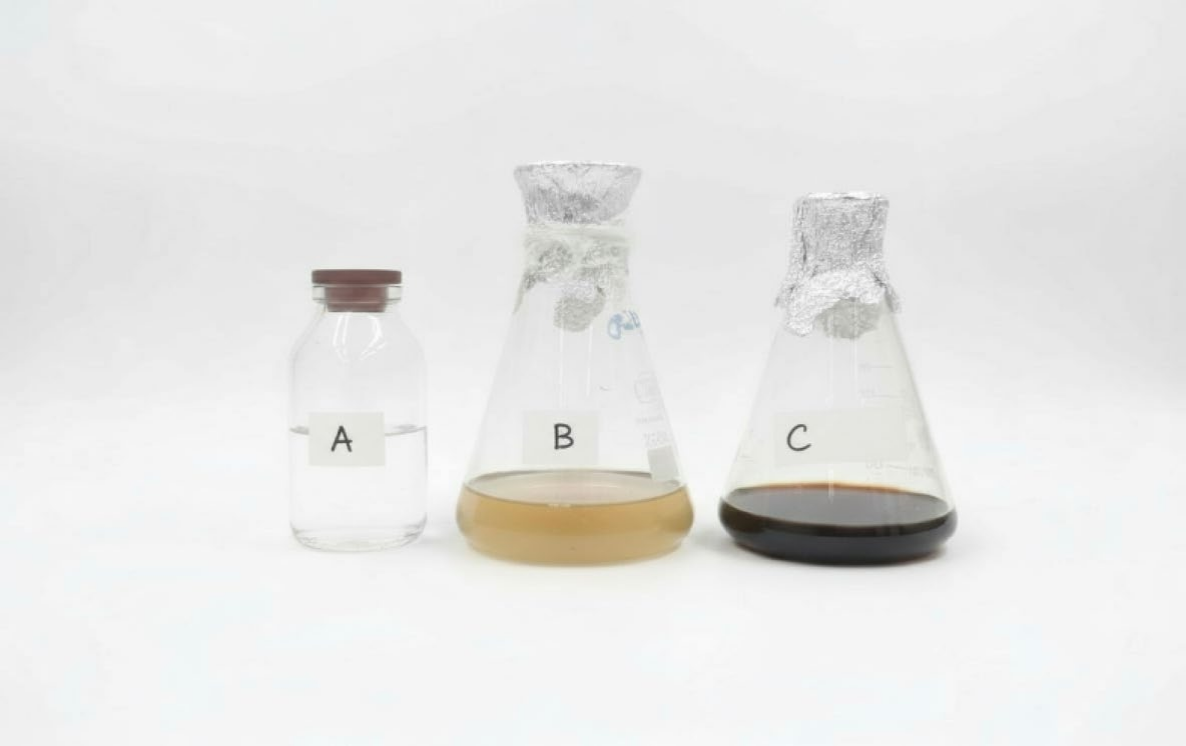
The distinct color of bio-synthesized silver nanoparticle, where **A** AgNO₃ solution, **B** *T.* filtrate, **c** solution of bio-synthesized silver nanoparticle.

Upon the addition of the *T.* filtrate extract to the 1 mM AgNO₃ solution, a distinct color change from pale yellow to brown was observed, as shown in Fig. 5, which is a primary indicator of nanoparticle formation. Initially, the AgNO₃ solution appeared colorless, while the fungal filtrate exhibited a faint yellowish tone. Within the first 30–60 min of incubation, the reaction mixture gradually shifted from pale yellow to light brown. Over the next few hours, the color intensified, turning into a stable dark brown suspension, which remained unchanged upon prolonged incubation, this distinct color transition, absent in the control solutions (AgNO₃ alone or fungal extract alone), strongly suggests the reduction of Ag^+^ ions to metallic Ag⁰ nanoparticles mediated by extracellular metabolites secreted by *Trichoderma* The intensity of the color is directly correlated with the concentration and density of the nanoparticles formed in the colloidal suspension.

### Characterization of silver nanoparticles

#### UV–visible spectroscopy

**Figure 6:**
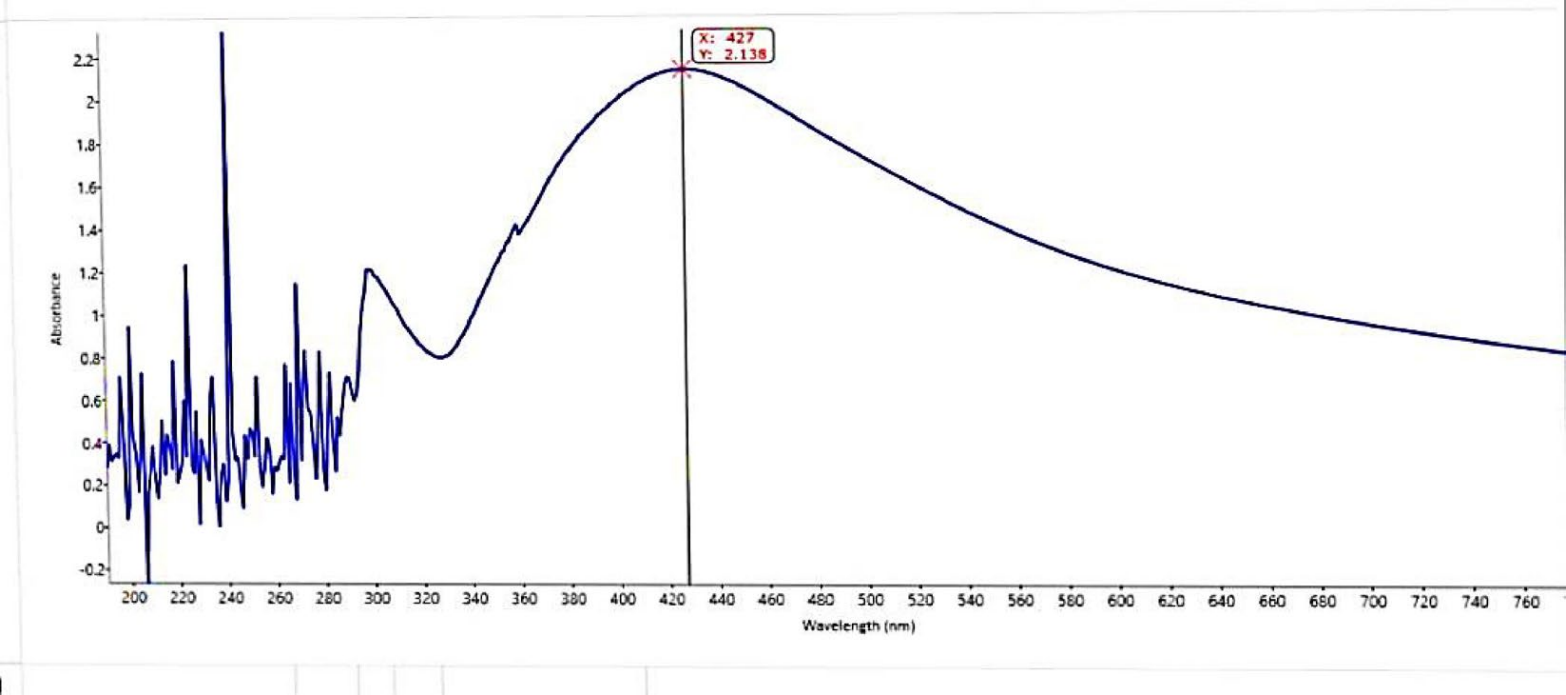
UV–Vis spectrum of the biosynthesized silver nanoparticles**.**

The UV–Vis spectrum of the biosynthesized AgNPs exhibited a characteristic surface plasmon resonance (SPR) peak centered at ~ 427 nm, as shown in (Fig. 6), confirming the formation of silver nanoparticles. The observed redshift and relatively broad peak suggest a moderate particle-size distribution and organic capping by biomolecules from the biological extract of *Trichoderma asperelloides*.

#### Transmission electron microscopy (TEM)

**Figure 7:**
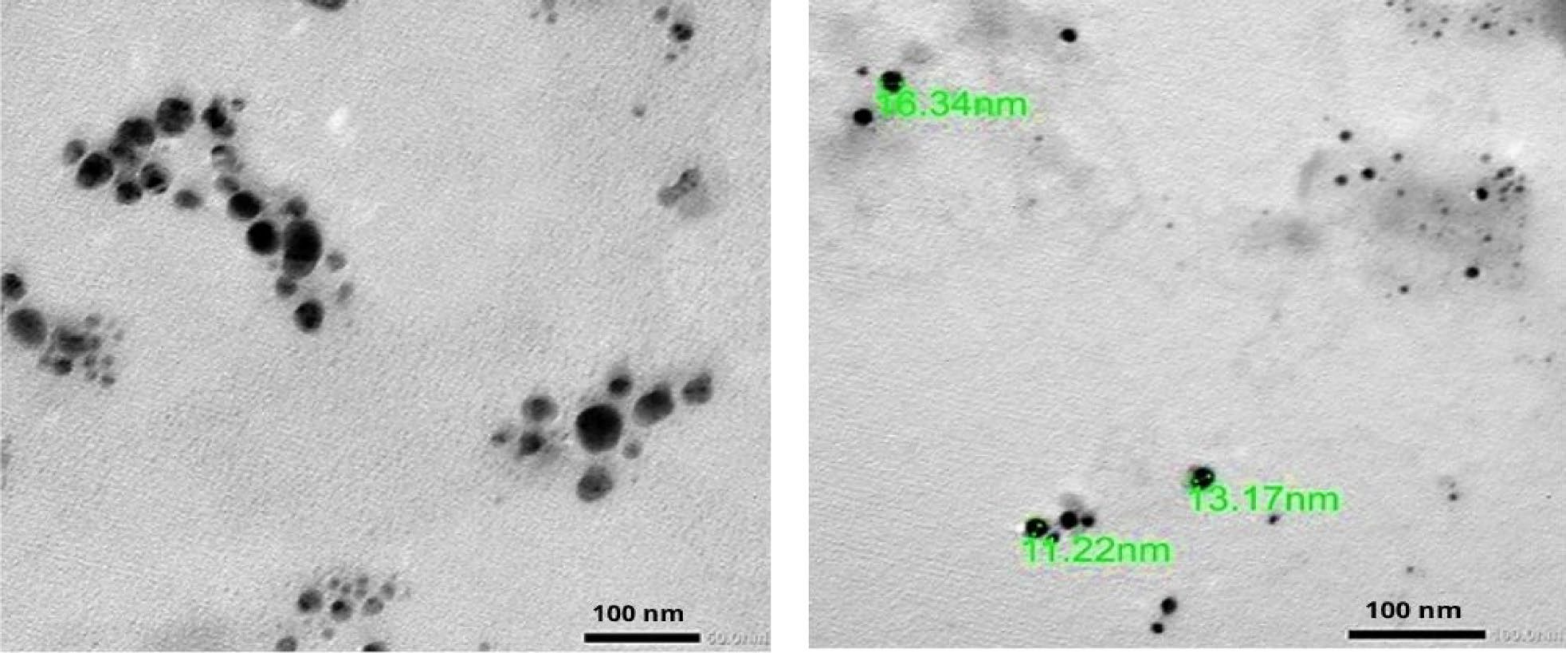
TEM micrograph of biosynthesized silver nanoparticles showing predominantly spherical to quasi-spherical particles with representative particle diameters of approximately 8.34–13.17 nm and an estimated mean size of 10.91 ± 2.45 nm.

TEM images of the biosynthesized AgNPs showed predominantly spherical to quasi-spherical particles with nanoscale dimensions. Representative particle measurements obtained from the TEM micrograph indicated particle diameters of approximately 8.34, 11.22, and 13.17 nm, corresponding to an estimated size range of 8–13 nm, with an approximate mean particle diameter of 10.91 ± 2.45 nm. These measurements support the nanoscale morphology of the synthesized AgNPs. However, a complete particle-size distribution histogram based on a larger particle population was not generated in the present study; therefore, the reported values represent representative TEM measurements rather than a full statistical size-distribution analysis (Fig. 7).

#### X-ray diffraction

**Figure 8:**
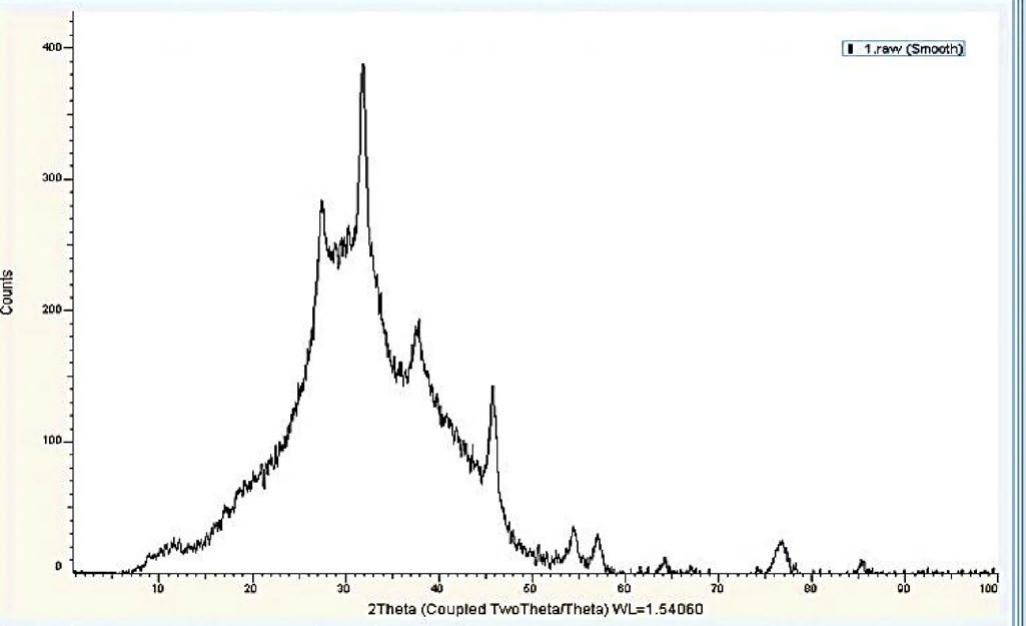
X-ray diffraction of the biosynthesized silver nanoparticles.

The XRD analysis of the biosynthesized silver nanoparticles (AgNPs) exhibited distinct diffraction peaks at approximately 2θ values of 38°, 44°, 64°, and 77° as shown in (Fig. 8). These peaks correspond to the (111), (200), (220), and (311) crystallographic planes of face-centered cubic (FCC) silver (Ag⁰), the presence of these characteristic peaks confirms the formation of crystalline metallic silver nanoparticles. The sharpness and intensity of the diffraction peaks indicate a high degree of crystallinity in the nanoparticles, while the absence of significant impurity peaks suggests the sample’s high purity. The average crystallite size, estimated by the Scherrer equation, is (10–30 nm), which is in accordance with the TEM observations in this study (~ 11.6 nm average size).

#### Scanning electron microscopy (SEM)

**Figure 9:**
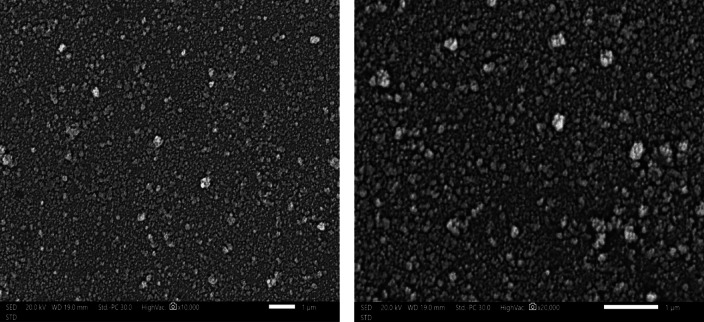
SEM images of the biosynthesized silver nanoparticles.

As indicated by the SEM micrographs (Fig. 9), most nanoparticles exhibited a predominantly spherical morphology and were largely monodisperse, with only limited aggregation. The measured particle diameters ranged from 6.6 to 17.2 nm. Some variability in nanoparticle size and, to a lesser extent, shape is expected for materials produced via biologically mediated synthesis routes.

#### EDX spectrum

**Figure 10:**
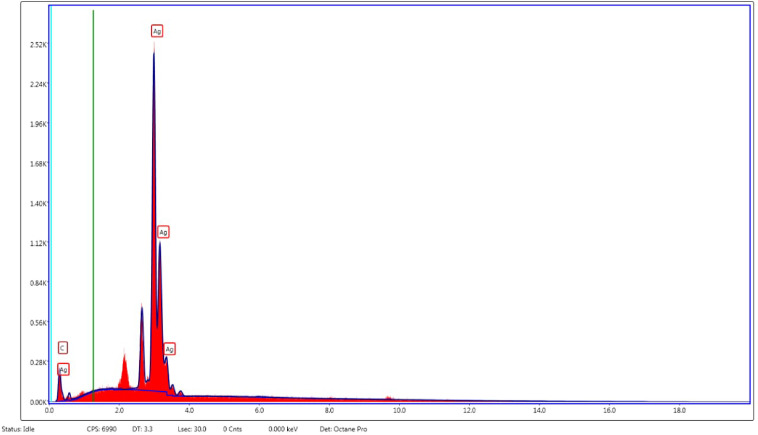
EDX spectrum of biosynthesized silver nanoparticles.

The energy-dispersive X-ray spectroscopy (EDX) spectrum of biosynthesized silver nanoparticles shows a dominant Ag peak at ~ 3.0 keV (Fig. 10), confirming the presence of metallic silver. Minor signals (e.g., C) correspond to organic capping agents derived from *Trichoderma* filtrate.

#### The Fourier transform infrared spectroscopy (FTIR) spectrum

**Figure 11:**
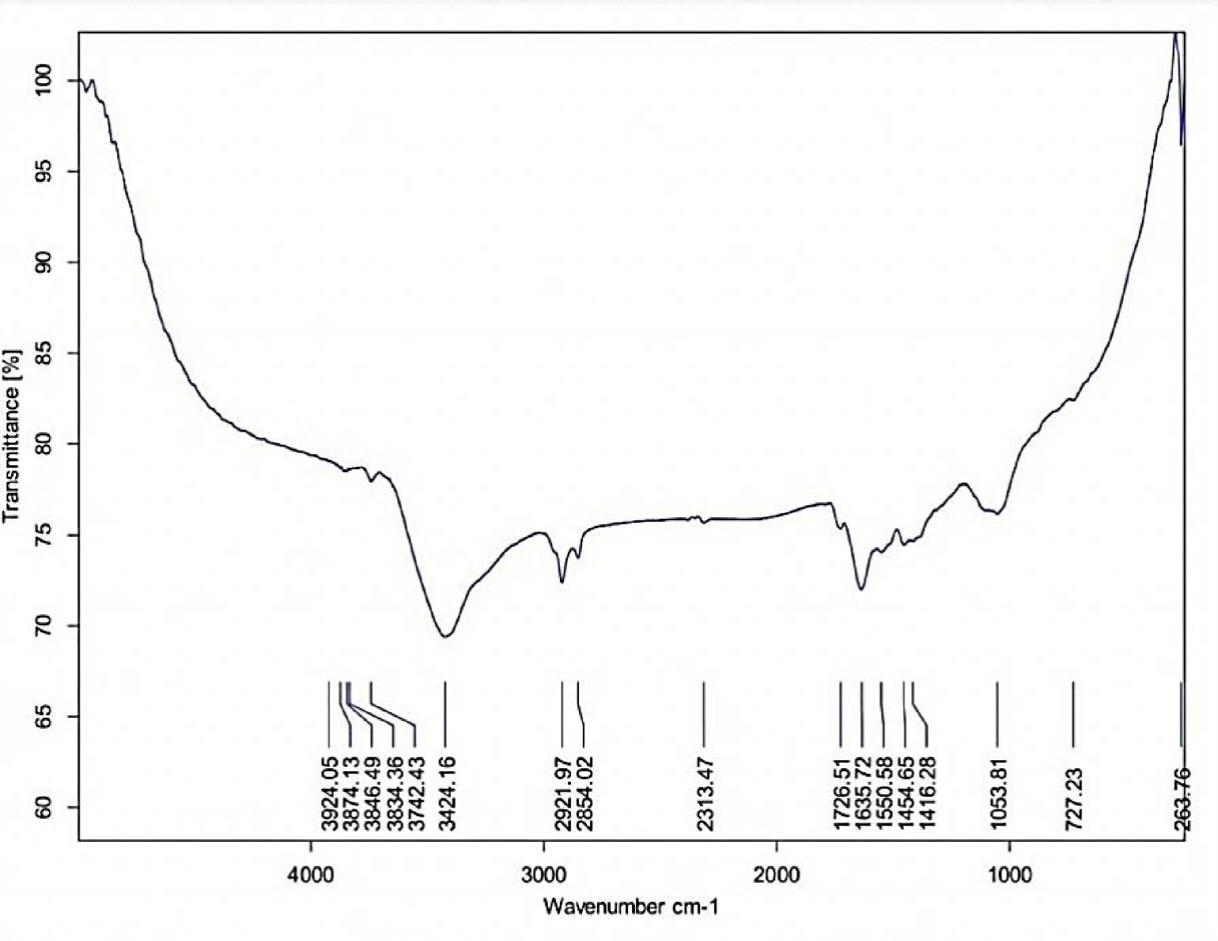
FTIR spectrum of the biologically synthesized silver nanoparticles.

The FTIR spectrum displayed absorption bands characteristic of fungal biomolecules involved in Ag^+^ reduction and nanoparticle stabilization. The broad O–H and N–H bands indicate the presence of hydroxyl and amino groups, whereas C–H, carbonyl, amide I and II, and C–O bands suggest the contribution of proteins, polysaccharides, and other fungal metabolites. These functional groups may participate in Ag^+^ reduction and act as capping agents via Ag–O and Ag–N interactions, thereby stabilizing biosynthesized AgNPs (Fig. 11).

#### Zeta potential analysis

**Figure 12:**
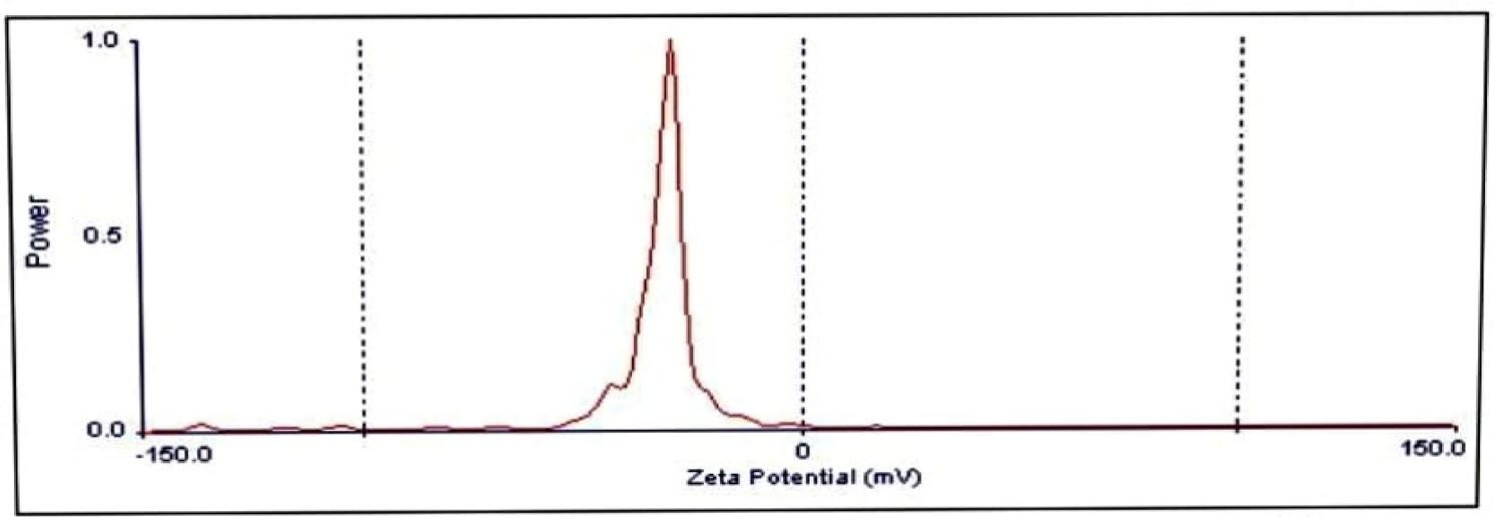
Zeta potential analysis of the biosynthesized silver nanoparticles.

The zeta potential analysis of the biosynthesized silver nanoparticles (AgNPs) revealed an average value of –30.05 mV with a particle size of approximately 40 nm at pH 5.6, as shown in (Fig. 12) and (Table 2), which can be regarded as moderately to highly stable AgNPs. The negative value indicates good colloidal stability due to charge repulsion among nanoparticles.

**Table 2:** Zeta potential measurements for biosynthesized silver nanoparticles.

Run	Zeta potential (mV)	Half width (mV)	Data retention (%)
1	− 19.15	2.69	75
2	− 27.07	4.45	50
3	− 22.37	5.19	60
4	− 34.70	5.18	75
5	− 31.45	3.86	60
6	− 36.12	3.88	60
7	− 38.25	5.50	75
8	− 32.78	5.60	75
9	− 28.57	3.97	75
10	− 30.07	2.97	75
Mean ± SD	− 30.05 ± 6.00	4.33 ± 1.03	68.00 ± 9.49

### Characterization of silver nanoparticles loaded on chitosan

#### UV–visible spectroscopy

**Figure 13:**
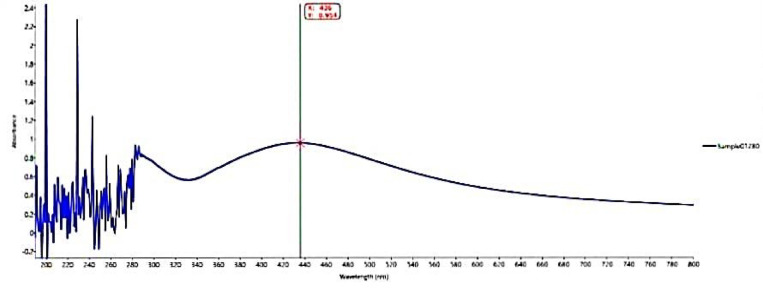
UV–Vis spectrum of the biosynthesized silver nanoparticles loaded on chitosan.

The UV–Vis spectrum confirms the successful biosynthesis and loading of AgNPs onto chitosan. The presence of the SPR band at 436 nm, as shown in (Fig. 13), demonstrates not only nanoparticle formation but also effective capping and stabilization by both chitosan and fungal metabolites. These results are consistent with reports showing that biopolymers such as chitosan act as stabilizing agents, shifting the SPR band slightly to longer wavelengths due to strong polymer–metal interactions.

#### X-ray diffraction

**Figure 14:**
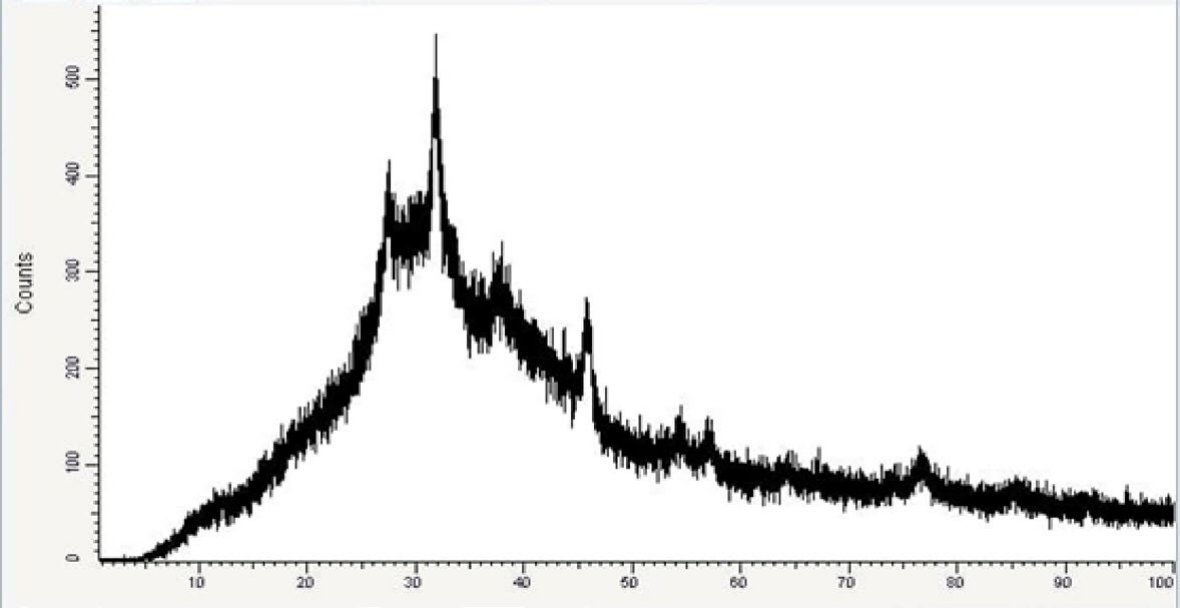
X-ray diffraction of the biosynthesized silver nanoparticles loaded on chitosan.

X-ray diffraction analysis of AgNPs loaded on chitosan showed a prominent diffraction peak at approximately 2θ = 31°, which may be attributed to the semi-crystalline nature of the chitosan matrix. Additional peaks at approximately 20 = 38° and 44° correspond to the (111) and (200) crystallographic planes of face-centered cubic metallic silver (Ag⁰), confirming the crystalline nature of the incorporated AgNPs. The broad background observed between 20° and 30° further supports the contribution of the amorphous chitosan matrix. Overall, the XRD pattern confirms the successful formation of crystalline AgNPs and their integration within the chitosan biopolymer support (Fig. 14).

#### Transmission electron microscopy (TEM)

**Figure 15:**
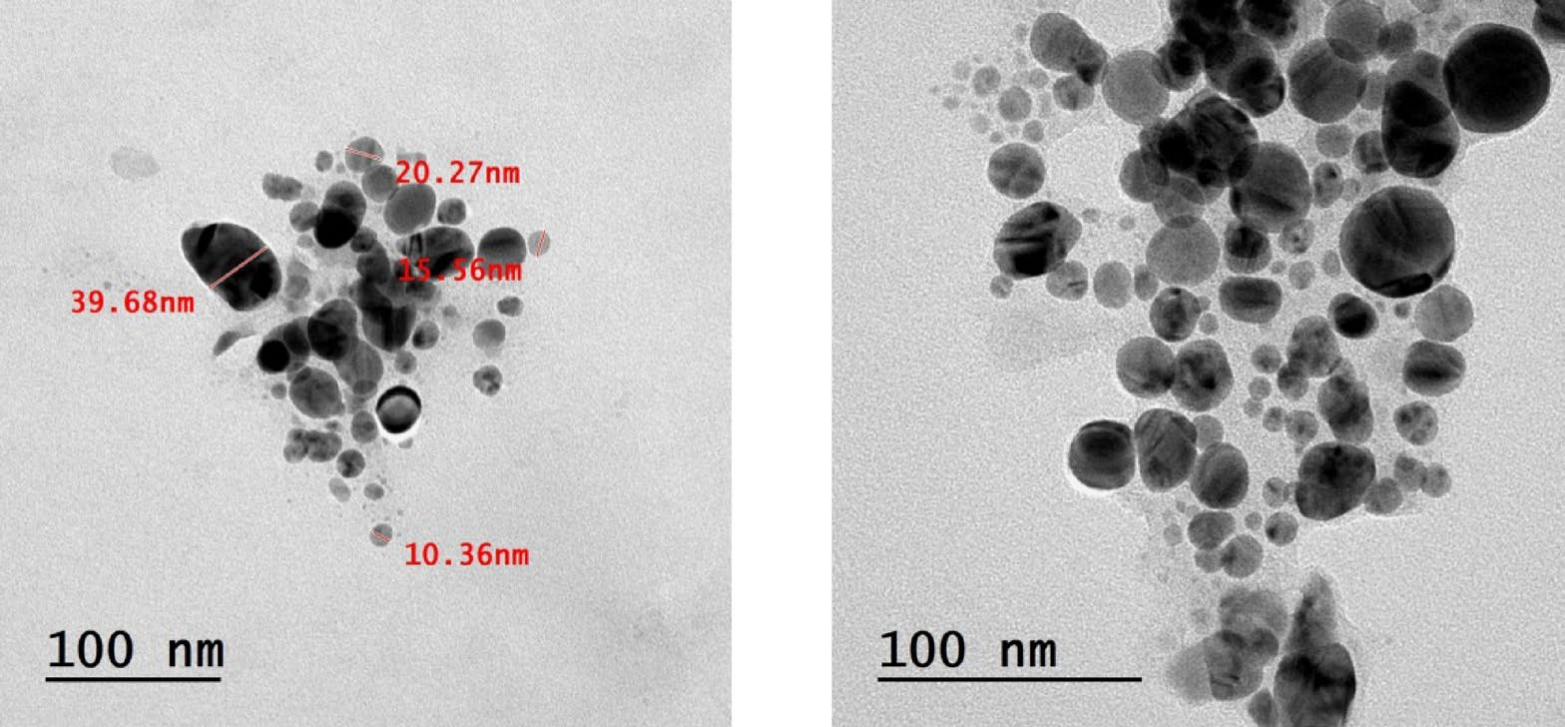
TEM images of the biosynthesized silver nanoparticles loaded on chitosan.

TEM images of the biosynthesized silver nanoparticles loaded on chitosan showed predominantly quasi-spherical to spherical metallic cores with a relatively broad size distribution. The measured particle diameters in the representative field ranged from approximately 10 to 40 nm, with representative values of 10.4, 15.6, 20.3, and 39.7 nm, and an average particle size of approximately 21 nm for the particles measured in this micrograph (Fig. 15). The metallic cores appeared as high-contrast electron-dense regions, often surrounded by a lower-contrast halo, consistent with a thin organic/biopolymer coating composed of chitosan and adsorbed fungal biomolecules. This coating may help stabilize nanoparticles. The particles were moderately well dispersed, although occasional close contacts and small clusters were observed, likely due to drying effects and polymer-mediated bridging during specimen preparation. No clear lattice fringes were observed in this image, preventing direct measurement of interplanar spacings. To provide a more quantitative assessment of nanoparticle morphology, particle-size distribution was evaluated from representative TEM/SEM micrographs and used to generate size-distribution histograms, allowing estimation of the dominant size range and average particle diameter.

#### Scanning electron microscopy (SEM)

**Figure 16:**
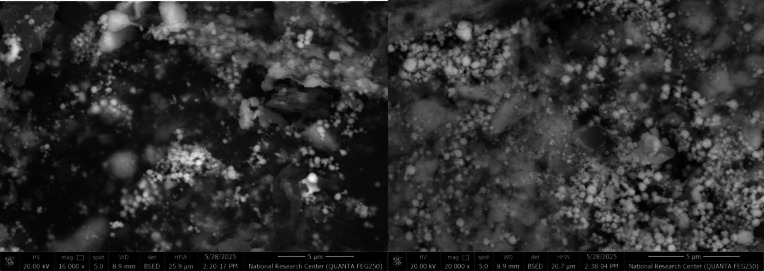
SEM images of the biosynthesized silver nanoparticles loaded on chitosan.

The SEM micrograph of the biosynthesized silver nanoparticles loaded onto chitosan revealed a heterogeneous but well-defined surface morphology, where numerous bright, spherical to nearly spherical nanoparticles were observed to be uniformly distributed across the chitosan matrix, as shown in (Fig. 16). The silver nanoparticles appeared as high-contrast spots embedded within or attached to the polymer surface, indicating the successful loading and stabilization of AgNPs by chitosan. The polymer matrix itself exhibited an irregular, porous-like morphology, which provides anchoring sites that facilitate nanoparticle deposition and prevent excessive agglomeration. Although small clusters of nanoparticles were visible, the overall distribution suggests that chitosan effectively acted as a supporting scaffold and capping agent, enhancing both dispersion and stability of the AgNPs.

#### EDX spectrum

**Figure 17:**
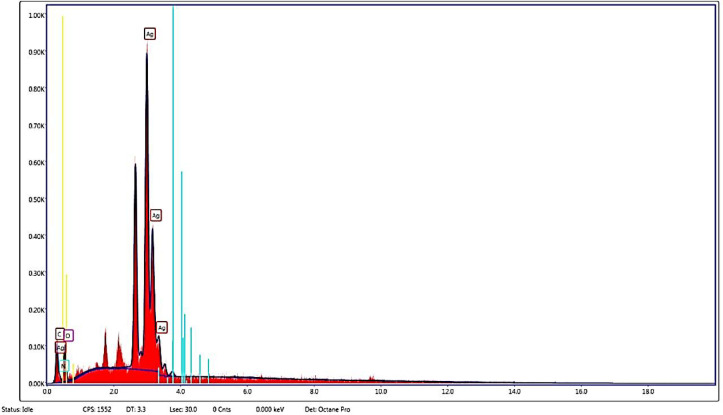
EDX spectrum of the biosynthesized silver nanoparticles loaded on chitosan.

The energy-dispersive X-ray spectroscopy (EDX) spectrum of the biosynthesized AgNPs–Chitosan sample showed intense, well-defined peaks for silver (Ag) at approximately 3 keV (Fig. 17), confirming the successful formation of metallic silver nanoparticles. In addition, characteristic signals of carbon (C) and oxygen (O) were detected, attributed to the chitosan matrix, which acts as a capping and stabilizing agent. The dominance of Ag peaks alongside the presence of C and O peaks clearly indicates the effective loading of silver nanoparticles onto the chitosan surface.

#### The Fourier transform infrared spectroscopy (FTIR) spectrum

**Figure 18:**
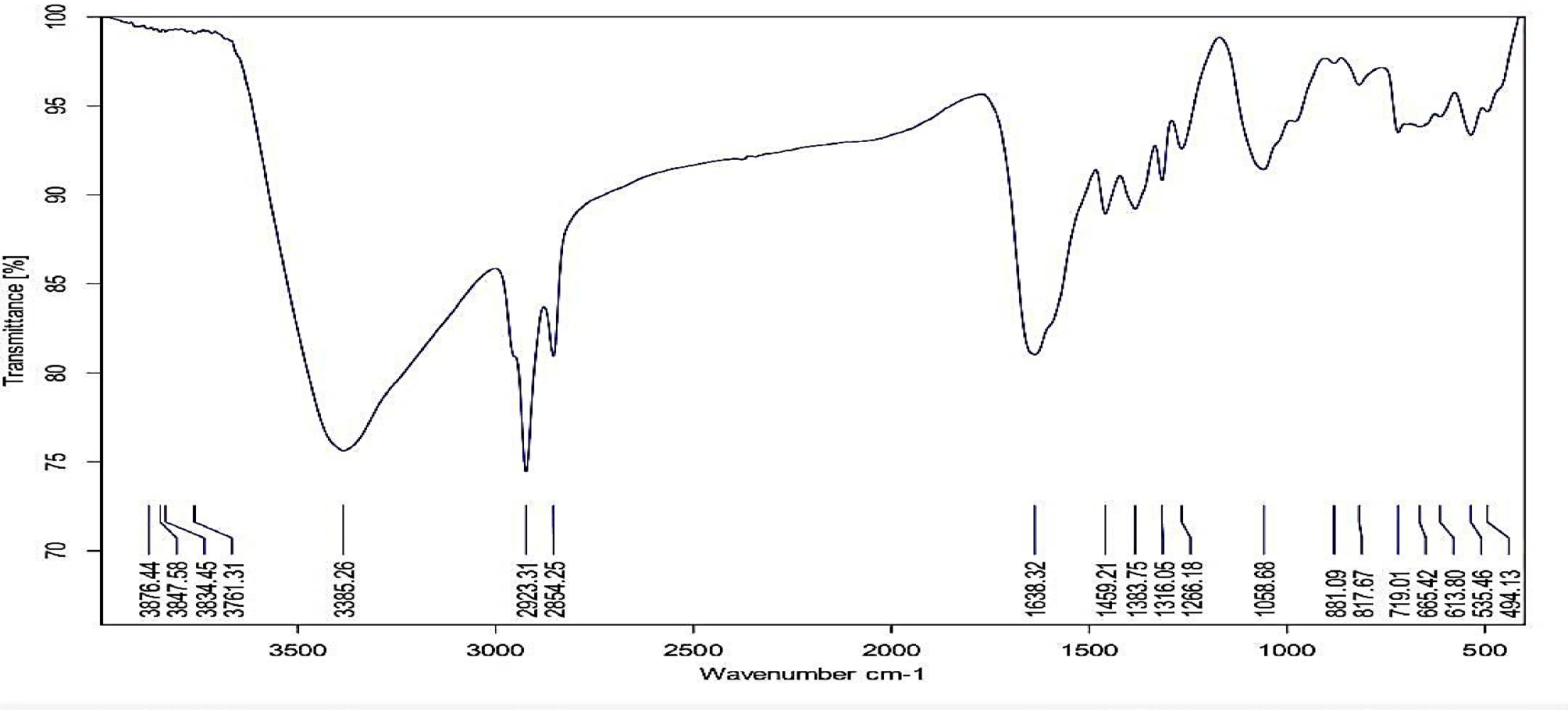
FTIR spectrum of the biosynthesized silver nanoparticles loaded on chitosan.

The FTIR spectrum of the AgNP–chitosan formulation indicated the successful incorporation of AgNPs into the chitosan matrix through interactions with functional groups present in chitosan and fungal biomolecules. Bands corresponding to O–H, N–H, C–H, carbonyl, amine, amide, and glycosidic/C–O groups suggest the involvement of these groups in nanoparticle binding, capping, and stabilization. In particular, the hydroxyl and amino groups of chitosan may contribute to polymer-mediated stabilization of the nanoparticles. Low-frequency bands in the range of 881–494 cm^−1^, assigned to Ag–O/Ag–N interactions, further support metal–ligand interactions and successful AgNP incorporation, as shown in Fig. 18. Overall, the FTIR analysis supports effective AgNP–chitosan interaction, with *Trichoderma*-derived biomolecules contributing to Ag^+^ reduction and nanoparticle stabilization.

#### Zeta potential analysis

**Figure 19:**
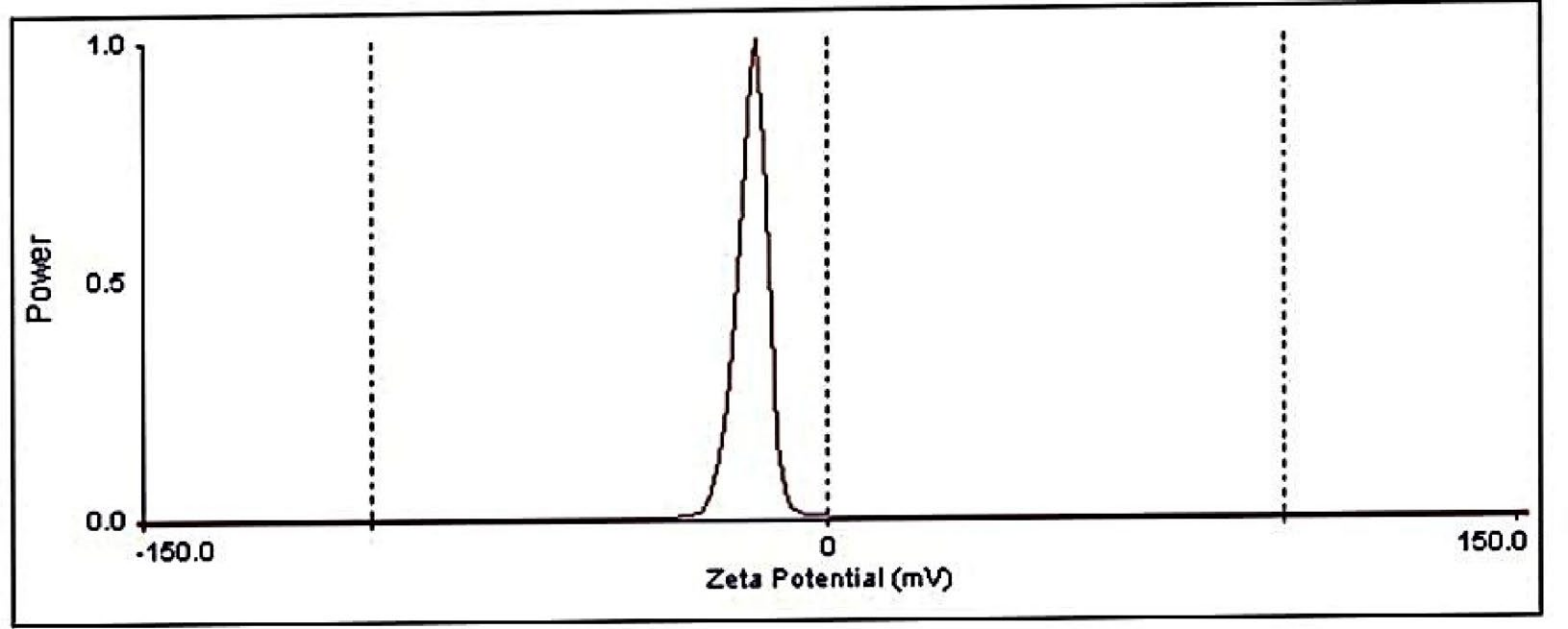
Zeta potential of the biosynthesized silver nanoparticles loaded on chitosan.

The zeta potential of the silver nanoparticles loaded on chitosan (AgNPs–Chitosan) was found to be –16.54 mV, with an average particle size of 40 nm at pH 5.6 as shown in (Fig. 19) and (Table 3). Although this value falls below the ± 20 mV threshold generally considered necessary for strong electrostatic stabilization, the incorporation of chitosan provides an alternative stabilization mechanism. Specifically, chitosan acts as a polymeric capping agent, introducing steric stabilization in addition to its partial electrostatic contribution. This explains why, despite the lower zeta potential, the AgNPs–Chitosan system can still maintain colloidal stability**.**

**Table 3:** The results of zeta potential measurements for 10 replicates of biosynthesized silver nanoparticles loaded onto chitosan.

Run	Zeta potential (mV)	Half width (mV)	Data retention (%)
1	− 16.08	4.03	75
2	− 19.04	5.89	100
3	− 16.07	4.10	75
4	− 19.54	3.94	100
5	− 16.07	3.24	100
6	− 13.11	3.85	50
7	− 17.07	5.54	100
8	− 17.06	2.99	75
9	− 19.32	3.54	100
10	− 15.09	3.30	43
Mean ± SD	− 16.84 ± 2.03	4.04 ± 0.96	81.80 ± 21.89

### Physico‑biochemical and molecular characteristics of infected Onion plants under the effect of biological control treatment

#### Effect of different treatments on the plant oxidative enzymes

**Figure 20:**
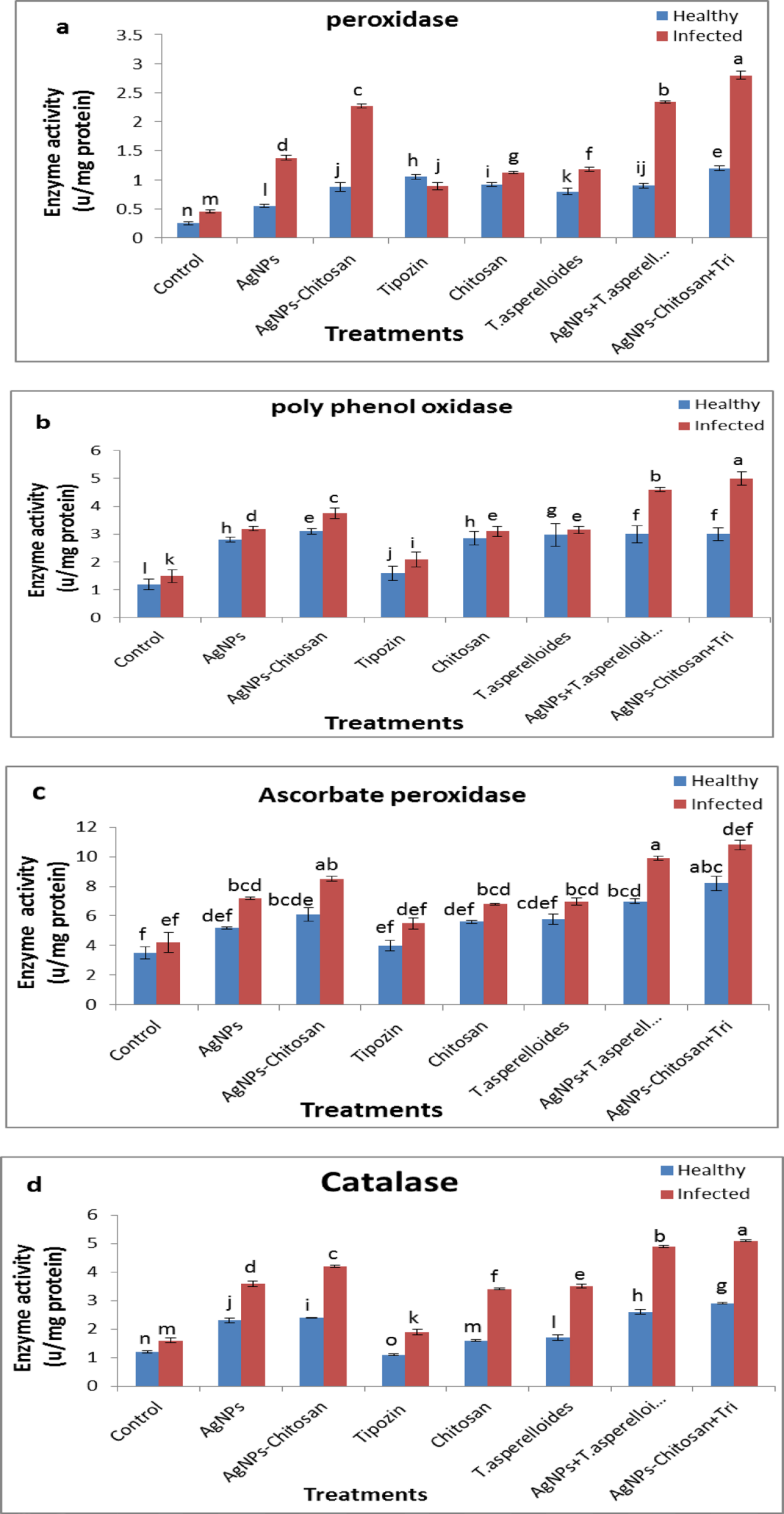
Activity of plant oxidative enzymes: peroxidase (**a**), polyphenol oxidase (**b**), ascorbate peroxidase (**c**), and catalase (**d**) for both infected and non-infected onion plants using different treatments. Different letters on columns revealed the significant variations measured by Duncan’s multiple range test at p < 0.05. (AgNPs)Silver nanoparticles (T. asperelloide ); *T. asperelloide****.***

There is a noticeable increase in peroxidase, polyphenol oxidase, ascorbate peroxidase and catalase activity in all infected onion plants compared to the non-infected ones. Among the different treatments, treated plants with AgNPs–Chitosan* T. asperelloide* (AgNPs–CHN–*T. asperelloide* ) exhibited the highest activity of the tested oxidative enzymes (2.8, 5, 10.8, 5.1 U/mg protein), respectively, as illustrated in (Fig. 20a–d), followed by AgNPs–*T. asperelloide* and AgNPs–CHN. These treatments were significantly more effective than the individual applications of AgNPs, chitosan, or *T. asperelloide* alone. Moderate increases in the activity of the tested enzymes were recorded in plants treated with chitosan, fungicide, or *T. asperelloide*, suggesting their partial ability to mitigate oxidative stress caused by fungal infection. The infected control showed the lowest measured oxidative enzyme activity (0.45, 1.5, 4.21, 1.6 u/mg protein, respectively). The highest enzyme activity has been shown in ascorbate peroxidase compared to other enzymes.

#### Effect of different treatments on non-enzymatic antioxidants

**Figure 21:**
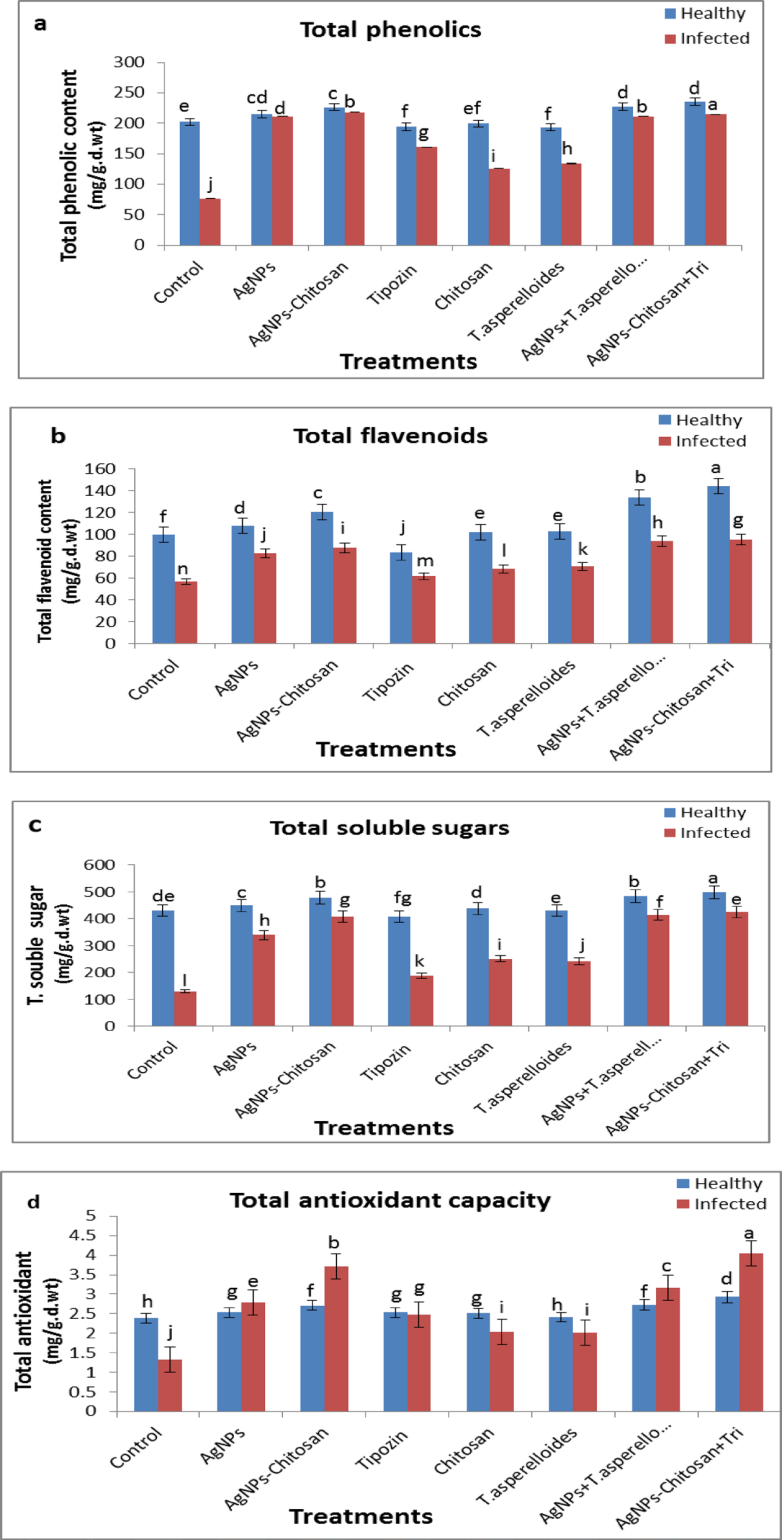
Effect of different treatments on the total phenolic content (**a**), total flavonoid content (**b**), total soluble sugar (**c**), and total antioxidant capacity (**d**) of both infected and non-infected onion plants. Different letters on columns revealed the significant variations measured by Duncan’s multiple range test at p < 0.05. (AgNPs)Silver nanoparticles (*T. asperelloide* ); *T. asperelloide****.***

Figure 21a–c shows that all applied treatments markedly increased the total phenolic, flavonoid, and total soluble sugar contents in onion plants infected with *Sclerotium cepivorum* compared with the infected untreated control. Among the tested treatments, the combination treatments AgNPs–CHN–*T. asperelloide* and AgNPs–T. Asperelloide recorded the highest values of total phenolic, flavonoid, and soluble sugar contents (214.76, 94.985, 424.12 mg/g.d.wt) and (211.39, 93.66, 414.07 mg/g.d.wt) respectively, which were followed by treatment with AgNPs–CHN and AgNPs alone. Moderate increases were observed in plants treated with chitosan or fungicide or *T. asperelloide* separately, as they showed a noticeable but lower increase compared with the nanoparticle-based treatments (126.33, 68.03, 251.14 mg/g.d.wt),(160.86, 61.38, 187.92 mg/g.d.wt), and (133.91, 70.34, 241.34 mg/g.d.wt). In contrast, the infected untreated control exhibited the lowest phenolic, flavonoid, and soluble sugar contents (76, 56.43, and 129.61 mg/g.d.wt, respectively). In general, the non-infected plants exhibited higher levels of phenolics, flavonoids, and soluble sugars than the infected plants across all treatments.

The total antioxidant capacity TAC increased markedly in all infected onion plants compared to non-infected ones, as indicated in Fig. 21d. Among all treatments, the combination AgNPs–CHN–*T. asperelloide* exhibited the highest TAC value (4.03 mg/g.d.wt), followed by AgNPs–*T. asperelloide* and AgNPs–CHN (3.7 mg/g.d. wt) and (3.17 mg/g.d.wt) respectively. Fungicide showed moderate increases in TAC compared with the infected control (2.485 mg/g.d.wt). Separate treatments with chitosan and *T. asperelloide* recorded the lower TAC values relative to the combined nano-treatments, but still higher than the infected untreated control (2.04 mg/g.d.wt) and (2.06 mg/g.d.wt) respectively. The lowest TAC value was recorded in the infected untreated control plants (1.33 mg/g.d.wt).

#### Effect of different treatments on the oxidative stress markers

**Figure 22:**
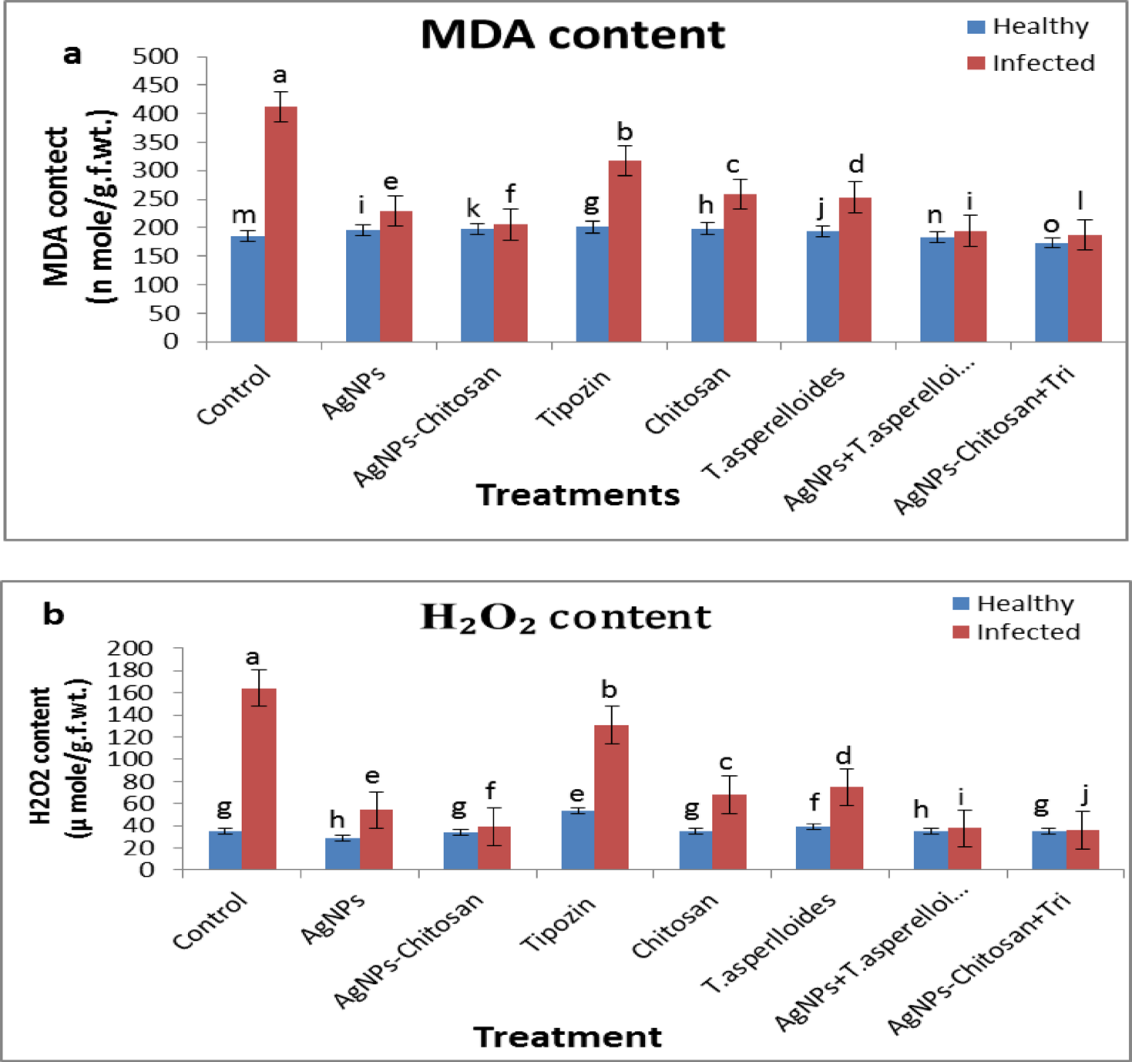
Effect of different treatments on Malondialdehyde content (MDA) (**a**) and H₂O₂ content (**b**) between infected and non-infected onion plants. Different letters on columns revealed the significant variations measured by Duncan’s multiple range test at p < 0.05. (AgNPs)Silver nanoparticles (T. asperelloide );*T. asperelloide* (MDA); Malondialdehyde (H₂O₂);Hydrogen-

Figure 22a illustrates the Malondialdehyde content, which was measured in onion plants infected with *S. cepivorum* and treated with various agents. Infected control plants show the highest levels of MDA value (412 nmol/g.f.wt.). Plants treated with AgNPs-CHN-*T. asperelloide* and AgNPs-*T. asperelloide* displayed a marked reduction in MDA levels compared to the control. Combined treatments of AgNPs-CHN revealed lower MDA values of about (205.3nmole/g.f.wt.). Treated plants with chitosan, a fungicide, or T. asperelloide separately reduced MDA levels relative to the infected control. But treatments involving AgNPs-*T. asperelloide* or AgNPs-CHN-*T. asperelloide*, demonstrated a highly significant decrease in MDA levels relative to the infected untreated control(194.6, 187.7 nmole/g.f.wt.), respectively.

In Fig. 22b, the concentration of H₂O₂ was measured in onion plants infected with *S. cepivorum* and subjected to different treatments. Among the infected samples, the untreated control exhibited the highest H₂O₂ content (164.22 µ mole/g.f.wt.). Treatment with AgNPs led to a marked decrease in H₂O₂ accumulation (54.18 µmole/g.f.wt). Plants treated with AgNPs-CHN showed a significant reduction in H₂O₂ content (39.34 µmole/g.f.wt.). In comparison, both chitosan and fungicide treatments resulted in moderate declines in H₂O₂ levels relative to the infected control (68.14 µmole/g.f.wt.) and (130.76 µmole/g.f.wt.), respectively. The combinations of AgNPs-*T. asperelloide* and AgNPs-CHN-*T. asperelloide* exhibited the lowest H₂O₂ concentrations in infected plants (37.6, 36.24 µmole/g.f. wt) respectively.

#### Effect of different treatments on non-enzymatic antioxidants

**Figure 23:**
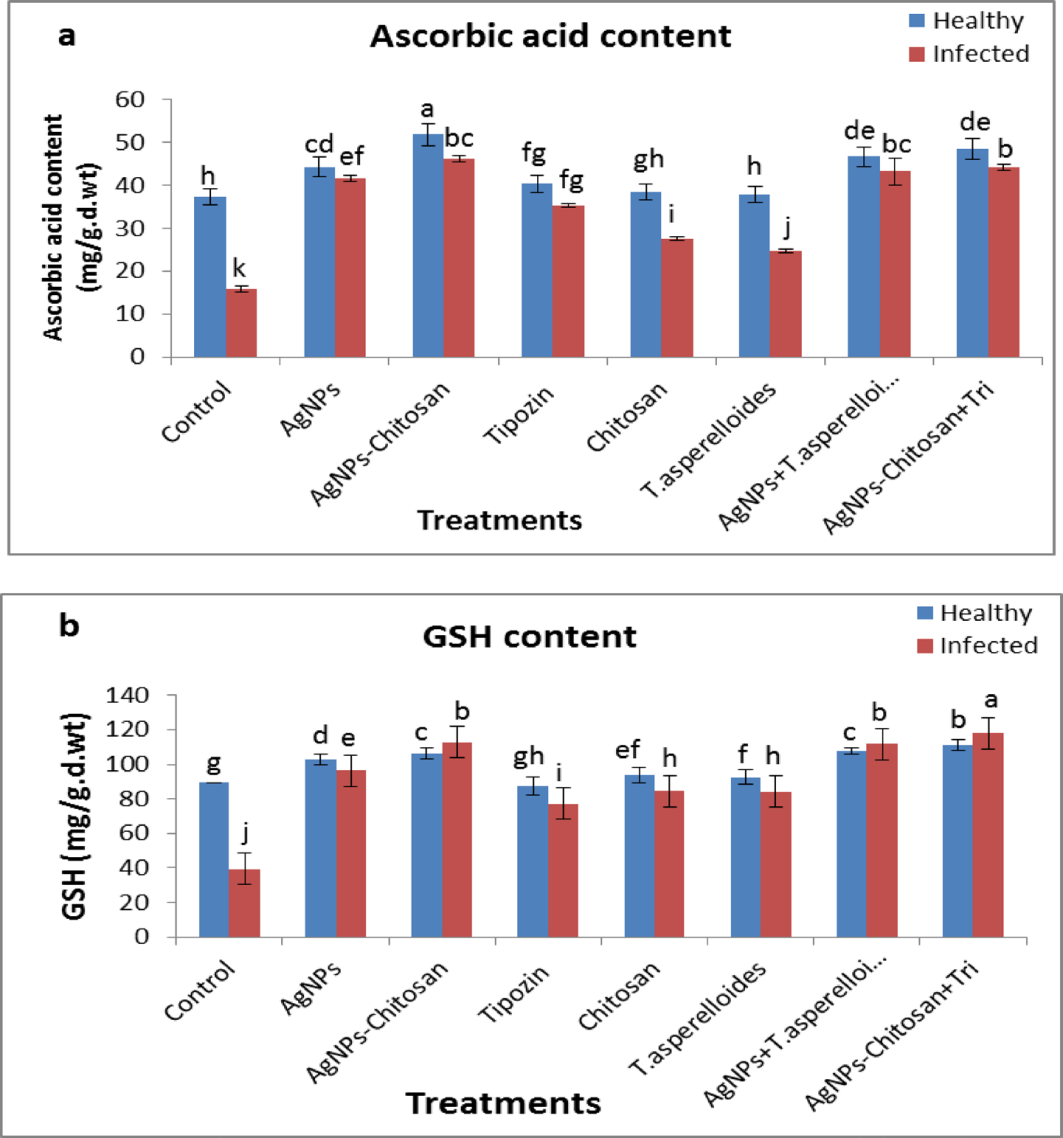
Effect of the different treatments on ascorbic acid content (**a**) and glutathione content (**b**) for both infected and non-infected onion plants. Different letters on columns revealed the significant variations measured by Duncan’s multiple range test at p < 0.05. (AgNPs); Silver nanoparticles (T. asperelloide ); *T. asperelloide* (GSH); glutathione.

Treatments of AgNPs-CHN, AgNPs + *T. asperelloide* , and AgNPs-CHN + T. asperelloide resulted in elevated ascorbic acid levels relative to infected control plants (46.10, 44.16, 43.13 mg/g.d.wt), respectively, as illustrated in Fig. 23a. Application of fungicide, chitosan, or *T. asperelloide* separately produced moderate increases (35.10, 27.59, 24.68 mg/g.d.wt), whereas the infected control plants maintained the lowest ascorbic acid content (15.76 mg/g.d.wt). Consistently non-infected plants showed higher ascorbic acid concentrations than infected plants, regardless of treatment.

Also, Fig. 23b shows variation in GSH content among treatments in both infected and non-infected plants. In general, non-infected plants showed slightly higher GSH levels than infected plants under most treatments. The highest GSH values were recorded in the AgNPs–CHN–T. asperelloide treatment(118.09 mg/g.d.wt), followed by AgNPs–T. asperelloide (111.61 mg/g.d.wt) and AgNPs-CHN.(106.38 mg/g.d.wt) Moderate levels of GSH were observed in plants treated with chitosan, fungicide, and* T. asperelloide*, whereas the infected control treatment showed the lowest GSH value (39.52 mg/g.d.wt).

#### Effect of different treatments on Photosynthetic pigments

**Figure 24:**
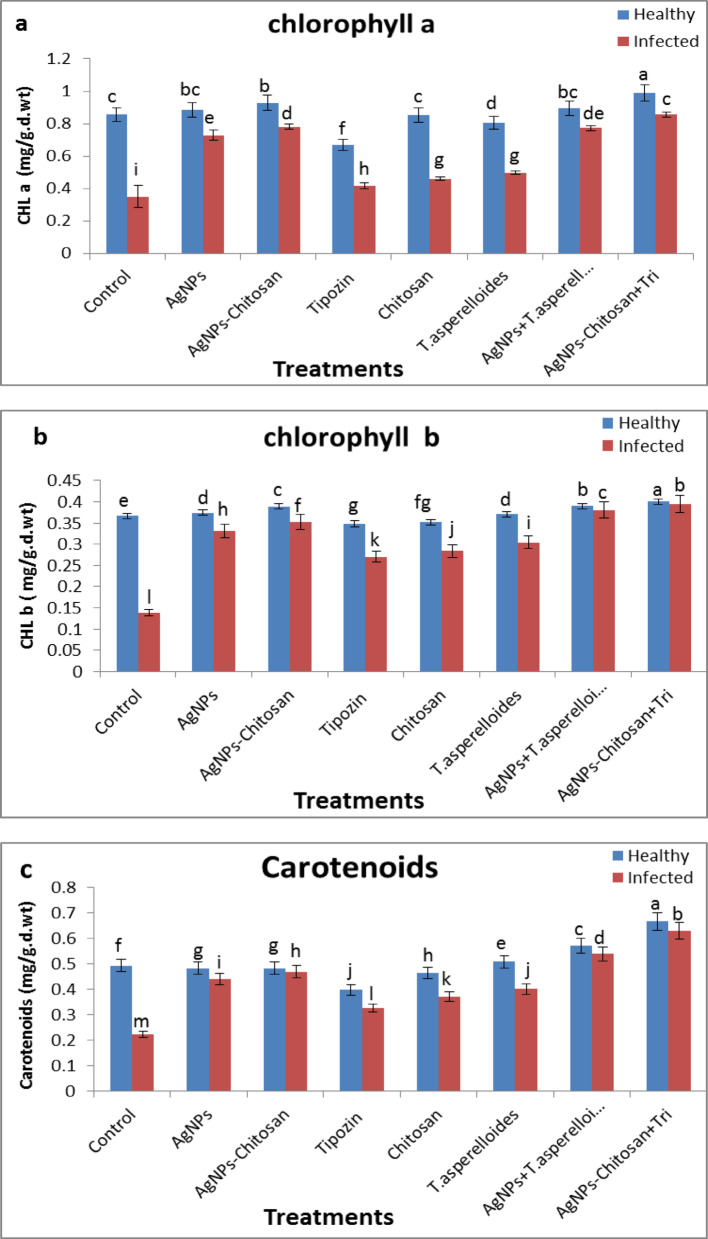
Photosynthetic pigments of onion plants: chlorophyll a (**a**), chlorophyll b (**b**), and carotenoid content( **c**). Different letters on columns revealed the significant variations measured by Duncan’s multiple range test at p < 0.05. (AgNPs) Silver nanoparticles (*T. asperelloide* ); *T. asperelloide .*

The results in Fig. 24a–c show that infection with *Sclerotium cepivorum* markedly reduced the chlorophyll a, chlorophyll b, and carotenoid contents in onion leaves compared with the corresponding non-infected plants. However, the extent of reduction varied among the different treatments. The AgNPs-CHN-T. Asperelloide treatment recorded the highest chlorophyll a, chlorophyll b, and carotenoid content in both infected and non-infected plants (0.85, 0.39, 0.63 mg/g.d.wt) and (0.99, 0.40, 0.66 mg/g.d.wt), respectively. The AgNPs-T. Asperelloide treatment also shows relatively high chlorophyll a, chlorophyll b, and carotenoid levels in infected plants (0.77, 0.38, 0.54 mg/g.d.wt, respectively). Treatments with T. asperelloideand chitosan individually exhibited moderate efficacy, with noticeably reduced chlorophyll a, chlorophyll b and carotenoid levels in infected plants compared to the combined treatments (0.49, 0.30, 0.40 mg/g.d.wt) and (0.46, 0.28, 0.37 mg/g.d.wt), respectively. The fungicide treatment resulted in lower chlorophyll a, chlorophyll b, and carotenoid content in infected plants compared to the bio-based treatments (0.41, 0.27, and 0.32 mg/g.d.wt, respectively). The infected control group exhibited the lowest photosynthetic pigment content (0.35, 0.13, 0.22 mg/g.d.wt), respectively.

#### Effect of different treatments on the plant growth parameters

**Figure 25:**
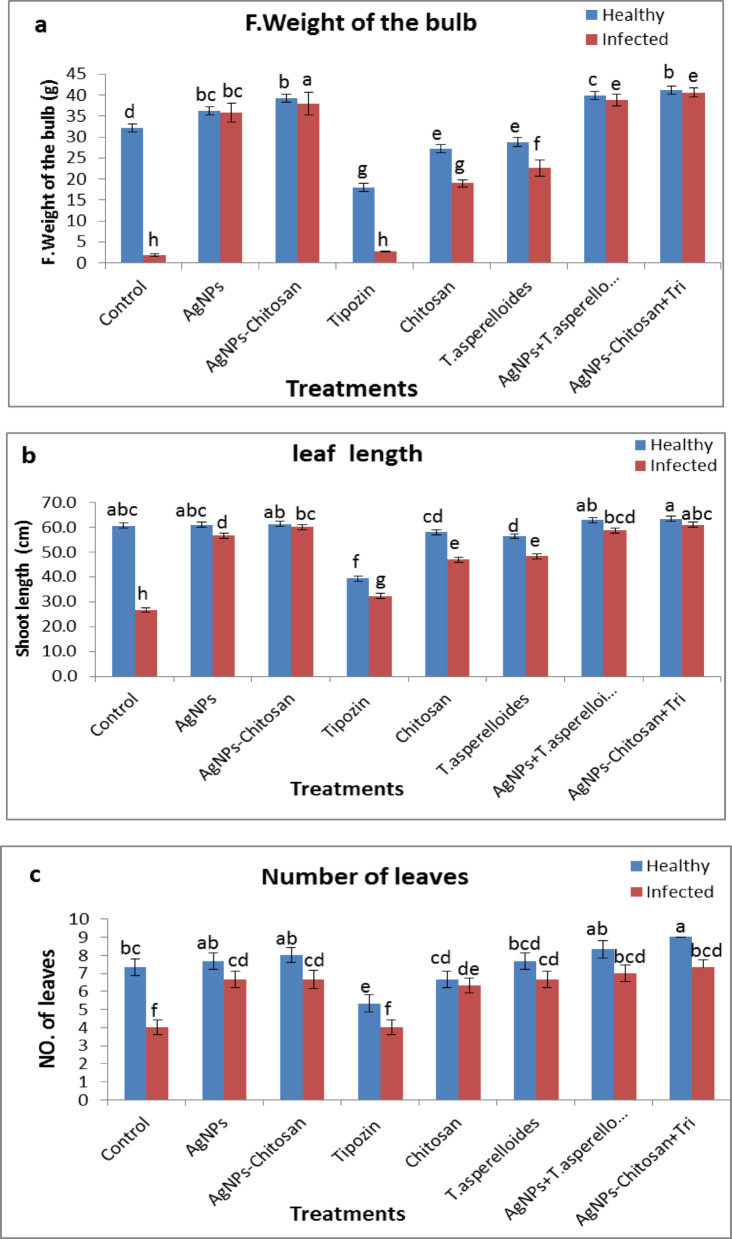
Growth parameter of onion plants: fresh weight of the bulbs (**a**), leaf length (**b**), and number of leaves (**c**). Different letters on columns revealed the significant variations measured by Duncan’s multiple range test at p < 0.05. (AgNPs) Silver nanoparticles (*T. asperelloide* ); *T. asperelloide*.

The weight of onion bulbs infected with *S. cepivorum* and subjected to various treatments showed substantial variation among the experimental groups, as indicated in Fig. 25a. Infected control plants displayed the lowest bulb weight (1.87 g). ​Compared with the healthy control (32.2 g), all treatments increased bulb weight. The most significant improvement was observed in plants treated with AgNPs-CHN-*T. asperelloide* , AgNPs-*T. Asperelloide and AgNPs-CHN also produced significantly higher bulb weights in the treated infected plants compared to the infected control plants (40.66, 38.82, 38.04 g),* respectively. Chitosan, *T. asperelloide, and fungicide treatments led to moderate increases in bulb weight, while AgNPs alone provided significant improvement (36.33 g) over the infected control but were less effective than the combined nano-biological treatments, as indicated in* Fig. 25a. Fungicide treatment was less effective than other strategies, as evidenced by lower bulb weight in infected plants (2.75 g).

As shown in Fig. 25b, the leaf length of tested onion plants infected with *S. cepivorum* and subjected to various treatments showed clear differences across experimental groups. The infected control plants exhibited the shortest leaf length (26.7 cm). In contrast, all treatments resulted in notable increases in leaf length relative to the control. The greatest enhancement was observed in plants treated with AgNPs-CHN-*T. asperelloide*, AgNPs-CHN and AgNPs-*T. asperelloide* (61,5, 60, 58.7 cm) respectively, where leaf length in infected plants closely approached or matched those in non-infected counterparts(63.3, 61.3, 62.8 cm). Chitosan and fungicide treatments also improved leaf length, though to a lesser extent compared to nano-biological combinations (47, 32.3 cm).

Figure 25c shows the number of leaves of onion plants infected with *S. cepivorum* and subjected to various treatments. The infected control group had the fewest leaves (4). In contrast, all treatment groups showed a marked increase in leaf number compared to the control. Among the treatments, the application of AgNPs-*T. asperelloide* and AgNPs-CHN-*T. asperelloide* followed by AgNPs-CHN, AgNPs resulted in greater numbers of leaves (9 8, 8, 7 leaves) compared to the fungicide, chitosan, or *T. asperelloide* alone. The fungicide, chitosan,* and T. asperelloide* treatments individually also led to a moderate increase in leaf number over the untreated (5, 6, 6 leaves), but were less effective than most nanomaterial-based combinations.

## Discussion

The present study aimed to control the persistent and destructive soil-borne fungus *S. cepivorum* using green silver nanoparticles (AgNPs) alone or loaded into a chitosan polymer. Additionally, *T. asperelloides*, a known antagonistic fungus, was evaluated for its ability to manage the pathogen.

Morphological characterization of the isolated pathogen revealed dense, white, cottony mycelial growth and the prolific formation of spherical to irregular sclerotia on PDA medium, which darkened with age. The progressive melanization of sclerotia is epidemiologically significant, as melanin accumulation enhances resistance to environmental stresses and microbial antagonism, thereby conferring aggressiveness and prolonged soil survival. These characteristics align with classical descriptions of *S. cepivorum*^49^.

Molecular identification of the pathogen using ITS-rDNA sequencing confirmed its identity as *S. cepivorum*, with 99–100% similarity to reference sequences in GenBank and clear phylogenetic clustering within the *S. cepivorum* clade. This underscores the utility of ITS regions as reliable fungal barcodes for accurate pathogen identification^17,18,50^.

The antagonistic fungus *T. asperelloides* exhibited highly branched, hyaline septate hyphae, with pyramidal or irregular conidiophore arrangements. The flask-shaped, lageniform phialides produced green, smooth-walled, subglobose conidia, forming compact clusters at the tips of the phialides. These morphological traits are consistent with previous descriptions^51^. Molecular confirmation via ITS sequencing validated the identity of *T. asperelloides*^17^.

Culture filtrate of *T. asperelloides* was used to biosynthesize AgNPs from silver nitrate. The pale-yellow mixture turned dark brown, indicating successful AgNP formation. Previous studies have reported the use of *Trichoderma* species for the synthesis of metallic nanoparticles, including silver nanoparticles^52,53^. Green synthesis offers advantages over chemical methods, such as reduced toxicity, enhanced biocompatibility, and improved nanoparticle stability^54^. Physicochemical characterization of the AgNPs formed in this study, using UV–Vis, XRD, FTIR, TEM, SEM, EDX, and zeta potential, aligned with previous reports^30,55,56^. The XRD peaks observed at approximately 2θ values of 38°, 44°, 64°, and 77° are consistent with the (111), (200), (220), and (311) planes of face-centered cubic metallic silver, supporting the crystalline nature of the biosynthesized AgNPs. FTIR analysis indicated the presence of hydroxyl, amino, carbonyl, amide, and C–O functional groups, suggesting the involvement of fungal biomolecules and chitosan in Ag^+^ reduction, nanoparticle capping, and stabilization. These interpretations support successful nanoparticle formation while remaining consistent with the structural and spectroscopic evidence obtained.

In our study, the incorporation of AgNPs into chitosan enhanced their stability, bioavailability, and antifungal efficacy against *S. cepivorum*, as supported by findings from El Hadrami et al.^57^ and Badawy and Rabea^58^.

Greenhouse experiments showed that all treatments significantly reduced onion white rot severity. The combination of AgNPs, chitosan, and *T. asperelloides* achieved the greatest disease suppression, suggesting a synergistic effect. The fungicide reference treatment (tebuconazole) served as a chemical control and improved disease suppression and plant growth parameters compared with the infected untreated control. However, its performance remained lower than that of the combined AgNPs chitosan* T. asperelloides* treatment, particularly regarding disease reduction, bulb fresh weight, and stress-related biochemical responses. These findings suggest that the integrated nano-biological formulation may provide broader protective effects than fungicide treatment alone under greenhouse conditions. This can be attributed to complementary mechanisms: mycoparasitism, enzymatic degradation, and nutrient competition by *T. asperelloides*; direct antifungal activity and oxidative stress induction by AgNPs; and membrane permeability modulation and defense induction by chitosan^8,55,58–60^. Combined treatments were more effective at controlling *S. cepivorum* compared to individual treatments^61^.

Infected onion plants exhibited reduced photosynthetic pigments, including chlorophyll a, chlorophyll b, and carotenoids^62^, as well as significant declines in total soluble sugars^63^, phenolic and flavonoid contents^57,64^, ascorbic acid (ASA), and reduced glutathione (GSH) levels^65,66^. Conversely, plants treated with AgNPs, chitosan, and *T. asperelloides* (individually or in combination) restored these parameters significantly^57,67^, including chlorophyll levels^67^, soluble sugars^68^, phenolics and flavonoids^63,69^, ascorbic acid, and GSH^45,65^. In contrast, hydrogen peroxide (H₂O₂) and malondialdehyde (MDA) levels were elevated in infected plants, indicating oxidative stress. These findings are consistent with previous studies demonstrating that *S. cepivorum* induces significant oxidative stress during host colonization^48^. Treatment with AgNPs, chitosan, and *T. asperelloides* significantly reduced H₂O₂ and MDA levels, with the combined treatments showing the greatest reductions, suggesting effective mitigation of oxidative damage and stabilization of cellular membranes, as previously observed^66,67^.

While infection induced a moderate increase in antioxidant enzyme activities, these responses were insufficient to counteract oxidative damage. In contrast, treated plants exhibited significantly higher activities of peroxidase (POD), ascorbate peroxidase (APX), glutathione peroxidase (GPX), and catalase (CAT). The combined treatments induced antioxidant defenses, as evidenced by elevated enzyme activities, which function synergistically to detoxify H₂O₂ and prevent accumulation of reactive oxygen species (ROS). Similar enzymatic upregulation has been documented in plants treated with *Trichoderma* spp. and chitosan-based formulations^64,66^. The enhanced antioxidant activity likely contributes to the suppression of *S. cepivorum* and to the induction of systemic plant immunity. In line with this, foliar application of chitosan nanoparticles in bananas enhanced defense responses and antioxidant enzyme activities while reducing Banana streak virus accumulation^60^.

In summary, the greenhouse results indicate that the combined application of AgNPs, chitosan, and *T. asperelloides* offers superior protection against onion white rot. This integrated strategy may target the pathogen while improving plant physiological performance and enhancing antioxidant and defense-related responses. This approach is consistent with Clarkson et al.^8^, who emphasized the need for integrated strategies for sustainable management of onion white rot. Similar findings in other crop–pathogen systems suggest that nanomaterials and chitosan can enhance host defenses and reduce disease development, including viral diseases such as Banana streak virus in banana^60^. For example, silver nanoparticles combined with chitosan upregulated the defense-related xylanase inhibitor (Xip-I) in wheat challenged with stem rust^70^. Additionally, micronutrient nanoparticles reduced peanut pod rots and aflatoxin contamination^71^, while chitosan induced systemic resistance against downy mildew in grapevine. Chitosan–copper nanoparticles also showed antifungal activity, supported by molecular docking analysis^71^. However, because the present findings were obtained under greenhouse conditions, field-level validation is required to confirm the efficacy, stability, and practical applicability of this nano-biological strategy under diverse environmental and soil conditions. Further work is also needed to evaluate the environmental safety of AgNP-based formulations. Future studies should assess AgNP persistence, dose-dependent toxicity, possible non-target effects, and impacts on soil microbial communities under field conditions before large-scale agricultural application.

The findings of this study highlight the potential of integrating biosynthesized AgNPs, chitosan, and *Trichoderma asperelloides* as a nano-biological strategy for sustainable management of onion white rot under greenhouse conditions. However, before large-scale agricultural application, further field validation is required to confirm treatment efficacy under variable environmental conditions. Future studies should also optimize application rates, clarify the molecular mechanisms underlying resistance, and assess AgNP persistence, long-term efficacy, and possible effects on non-target soil microbial communities. Because the present study was conducted under greenhouse conditions, further work is needed to evaluate the environmental safety of AgNP-based formulations. Future studies should assess AgNP persistence, dose-dependent toxicity, non-target effects, and possible impacts on soil microbial communities before broad field application.

## Conclusion

This study highlights the potential of integrating biosynthesized AgNPs, chitosan, and *Trichoderma asperelloides* as a nano-biological strategy for sustainable management of onion white rot under greenhouse conditions. The combined treatment provided the strongest disease suppression and improved plant physiological responses compared with individual treatments. Future studies should optimize application rates, assess AgNP persistence and environmental safety, and evaluate possible effects on non-target soil microbial communities. These findings are limited to greenhouse conditions and require field-scale validation before practical field recommendations can be made.

## Data Availability

The nucleotide sequences generated in this study were deposited in the NCBI GenBank database under accession numbers PX089568.1 for the pathogenic isolate (*Stromatinia cepivora* isolate S. c1) and PX107871.1 for the antagonistic isolate (*Trichoderma asperelloides* isolate 102).

## References

[1] Crowe, F. J. White rot of onion. In Compendium of Onion and Garlic Diseases (eds Schwartz, H. F. & Mohan, S. K.) 16–19 (APS Press, 2008).

[2] Entwistle, A. R. Allium white rot and its control (Horticultural Development Council, 1990).

[3] Abd El-Rahim, M. F. & Amein, A. M. Pathological studies on white rot disease of onion caused by Sclerotium cepivorum Berk. Egyptian Journal of Phytopathology 47, 1–15 (2019).

[4] Coleman, J. Biology and management of onion white rot (Sclerotium cepivorum). *Plant Dis.* **100**, 1461–1470 (2016).

[5] Coley-Smith, J. R., Parfitt, D. & Taylor, I. M. Studies on the survival of sclerotia of Sclerotium cepivorum Berk. *Plant. Pathol.* **39**, 58–68 (1990).

[6] Coley-Smith, J. R. & King, J. E. Germination of sclerotia of Sclerotium cepivorum stimulated by onion root exudates. *Ann. Appl. Biol.* **64**, 289–301 (1969).

[7] Clarkson, J. P., Payne, T., Mead, A. & Whipps, J. M. Effect of volatile sulphur compounds on sclerotial germination and mycelial growth of Sclerotium cepivorum. *Plant. Pathol.* **51**, 413–422 (2002).

[8] Clarkson, J. P. et al. Forecasting Sclerotium cepivorum white rot epidemics in onion crops. *Plant. Pathol.* **68**, 12–24 (2019).

[9] Sammour, R. H., Mahmoud, Y. A.-G., Mustafa, A.-Z. M. A. & Alhozeim, R. A. Effective and cheap methods to control Sclerotium cepivorum through using Clorox or sulfur powder and/or calcium oxide. *Res. J. Microbiol.* **6**, 902–911 (2011).

[10] Darwesh, O. M. & Elshahawy, I. E. Silver nanoparticles inactivate sclerotial formation in onion white rot disease. *Eur. J. Plant Pathol.* **160**, 917–934 (2021).

[11] Pung, H., Clarkson, J. P. & Whipps, J. M. Suicidal germination of Sclerotium cepivorum sclerotia induced by volatile sulfur compounds. *Crop Prot.* **143**, 105531 (2021).

[12] Hilton, S., Bennett, A. J., Whipps, J. M. & Clarkson, J. P. Biological control of Allium white rot (Sclerotium cepivorum): Current status and future prospects. *Biol. Control* **135**, 45–55 (2019).

[13] Mahmoud, Y. A.-G., Sammour, R. H., Mustafa, A.-Z. M. A. & Alhozeim, R. A. Role of sclerotia in the aggressiveness and pathogenicity of Sclerotium cepivorum. *Asian J. Microbiol. Biotechnol. Environ. Sci.* **23**, 16–23 (2021).

[14] Omar, H. S., Al Mutery, A., Osman, N. H., Reyad, N. E. & Abou-Zeid, M. A. Molecular marker analysis of stem and leaf rust resistance in Egyptian wheat genotypes and interpretation of the antifungal activity of chitosan-copper nanoparticles by molecular docking analysis. *PLoS ONE* **16**, e0257959. 10.1371/journal.pone.0257959 (2021). 34767570 10.1371/journal.pone.0257959 PMC8589204

[15] Atwa, A. A. et al. Leaf rust resistance in wheat and interpretation of the antifungal activity of silver and copper nanoparticles. *Sci. Rep.* **15**, 9429. 10.1038/s41598-025-91127-4 (2025). 40108210 10.1038/s41598-025-91127-4 PMC11923268

[16] Hussain, S. et al. Isolation, purification, and pathogenicity of Sclerotium cepivorum causing white rot of onion. *J. Plant Pathol.* **99**, 345–352 (2017).

[17] Ullah, I. & Chen, F. Exploring medicinal plants and their nutraceutical attributes from northern areas of Pakistan. *Food Sci. Appl. Microbiol. Rep.* **2**, 78–92 (2023).

[18] White, T. J., Bruns, T., Lee, S. & Taylor, J. Amplification and direct sequencing of fungal ribosomal RNA genes for phylogenetics. In PCR Protocols: A Guide to Methods and Applications (eds Innis, M. A., Gelfand, D. H., Sninsky, J. J. & White, T. J.) 315–322 (Academic Press, 1990).

[19] Tamura, K., Stecher, G. & Kumar, S. MEGA X: molecular evolutionary genetics analysis across computing platforms. *Mol. Biol. Evol.* **30**, 2725–2729 (2013). 29722887 10.1093/molbev/msy096 PMC5967553

[20] El-Debaiky, S. A. Antagonistic studies and hyphal interactions of the new antagonist Aspergillus piperis against some phytopathogenic fungi in vitro in comparison with Trichoderma harzianum. *Microb. Pathog.* **113**, 135–143 (2017). 29074431 10.1016/j.micpath.2017.10.041

[21] Bell, D. K., Wells, H. D. & Markham, C. R. In vitro antagonism of Trichoderma species against six fungal plant pathogens. *Phytopathology* **72**, 379–382 (1982).

[22] Domsch, K. H., Gams, W. & Anderson, T. H. *Compendium of Soil Fungi* (Academic Press, 1980).

[23] Watanabe, T. *Soil and Seed Fungi: Morphologies of Cultured Fungi and Key to Species* 2nd edn. (CRC Press, 1994).

[24] Raheem, A., Ahmad, M. & Khan, Z. Green synthesis of silver nanoparticles: mechanisms, applications and challenges. *J. Green Chem.* **21**, 345–367 (2025).

[25] Singh, P., Kim, Y. J., Zhang, D. & Yang, D. C. Biological synthesis of nanoparticles from plants and microorganisms. *Trends Biotechnol.* **34**, 588–599 (2016). 26944794 10.1016/j.tibtech.2016.02.006

[26] Al-Hazmi, F., Alghamdi, A. & El-Shall, M. S. Morphological and structural characterization of biogenic silver nanoparticles. *Mater. Sci. Eng. C* **146**, 113258 (2023).

[27] Bhat, R. et al. Microbial synthesis and characterization of metal nanoparticles. *Microb. Biotechnol.* **14**, 567–582 (2021).

[28] Scimeca, M., Bischetti, S., Lamsira, H. K., Bonfiglio, R. & Bonanno, E. Energy dispersive X-ray (EDX) microanalysis: a powerful tool in biomedical research and diagnosis. *Eur. J. Histochem.* **62**, 2841 (2018). 29569878 10.4081/ejh.2018.2841 PMC5907194

[29] Priyadarshini, E., Pradhan, N. & Sukla, L. B. X-ray diffraction and crystallite size analysis of biosynthesized silver nanoparticles. *Nano Struct. Nano Objects* **32**, 100926 (2025).

[30] Maheshkumar, A. et al. FTIR and spectral analysis of green-synthesized silver nanoparticles. *Nano Mater. Appl.* **9**, 104–119 (2025).

[31] Singh, R., Singh, S. & Prasad, S. M. Zeta potential and stability analysis of biologically synthesized nanoparticles. *Environ. Nanotechnol. Monit. Manag.* **20**, 100774 (2023).

[32] Demirbas, A. & Karsli, S. Chitosan-based nanocomposites for agricultural applications. *Int. J. Biol. Macromol.* **244**, 125432 (2025).

[33] Mahdy, A. M. M. & Aboelmagd, H. I. Soil infestation techniques for evaluating white rot disease management in onion. *Egypt. J. Plant Prot. Res.* **12**, 45–58 (2024).

[34] Metzner, H., Rau, H. & Senger, H. Untersuchungen zur Synchronisierbarkeit einzelner pigmentmangel-mutanten von Chlorella. *Planta* **65**, 186–194 (1965).

[35] Naguib, M. I., El-Shafey, H. M. & Khalil, S. I. Studies on the soluble carbohydrates of plants. *Physiol. Plant.* **21**, 1039–1047 (1968).

[36] Dubois, M., Gilles, K. A., Hamilton, J. K., Rebers, P. A. & Smith, F. Colorimetric method for determination of sugars and related substances. *Anal. Chem.* **28**, 350–356 (1956).

[37] Velikova, V., Yordanov, I. & Edreva,. Oxidative stress and some antioxidant systems in acid rain-treated bean plants. *Plant Sci.* **66**, 59–66 (2000).

[38] Heath, R. L. & Packer, L. Photoperoxidation in isolated chloroplasts: kinetics and stoichiometry of fatty acid peroxidation. *Arch. Biochem. Biophys.* **125**, 189–198 (1968). 5655425 10.1016/0003-9861(68)90654-1

[39] Oser, B. L. *Hawk’s Physiological Chemistry* 14th edn. (McGraw-Hill, 1965).

[40] Anderson, M. E. Determination of glutathione and glutathione disulfide in biological samples. *Methods Enzymol.* **113**, 548–555 (1985). 4088074 10.1016/s0076-6879(85)13073-9

[41] Jindal, K. K. & Singh, R. N. Phenolic content in male and female Carica papaya. *Physiol. Plant.* **33**, 104–107 (1975).

[42] Chang, C. C., Yang, M. H., Wen, H. M. & Chern, J. C. Estimation of total flavonoid content in propolis by two complementary colorimetric methods. *J. Food Drug Anal.* **10**, 178–182 (2002).

[43] El-Shora, H. M., El-Amier, Y. A. & Awad, M. H. Antioxidant enzymes and oxidative stress markers in plants. *J. Plant Biochem. Physiol.* **3**, 150 (2015).

[44] Kato, M. & Shimizu, S. Chlorophyll metabolism in higher plants. *Plant Cell Physiol.* **28**, 1293–1301 (1987).

[45] Nakano, Y. & Asada, K. Hydrogen peroxide is scavenged by ascorbate-specific peroxidase in spinach chloroplasts. *Plant Cell Physiol.* **22**, 867–880 (1981).

[46] Hopkins, F. G. & Tudhope, G. R. Glutathione peroxidase in plant tissues. *Biochem. J.* **134**, 429–436 (1973).

[47] Patterson, B. D., MacRae, E. A. & Ferguson, I. B. Estimation of hydrogen peroxide in plant extracts using titanium(IV). *Anal. Biochem.* **139**, 487–492 (1984). 6476384 10.1016/0003-2697(84)90039-3

[48] Steel, R. G. D., Torrie, J. H. & Dickey, D. A. *Principles and Procedures of Statistics: A Biometrical Approach* 3rd ed. (McGraw-Hill, 1997).

[49] Mahmoud, M., El Katatny, M. H. & Youssef, A. M. Morphological characterization and epidemiology of Sclerotium cepivorum in onion white rot. *Egypt. J. Crop Prot.* **19**, 80–96 (2021).

[50] Clarkson, J. P., McLean, M. N. & Ward, E. Molecular identification and phylogenetic analysis of Sclerotium cepivorum isolates. *Mycol. Res.* **123**, 345–352 (2019).

[51] Samuels, G. J. & Ismail, A. Taxonomy and morphological traits of Trichoderma species. *Fungal Divers.* **47**, 1–34 (2011).

[52] Singh, P. et al. Biosynthesis of silver nanoparticles using Trichoderma spp. *J. Nanobiotechnol.* **14**, 52 (2016).

[53] Bhat, R. et al. Nano biological generation via microbial processes. *Microb. Biotechnol.* **14**, 567–582 (2021).

[54] Raheem, A. Green synthesis and nanoparticle stability: a comparative review. *J. Green Chem.* **21**, 345–367 (2025).

[55] El Saadony, M. T. et al. Physicochemical characterization of biogenic silver nanoparticles. *J. Nanosci. Nanotechnol.* **22**, 4561–4579 (2022).

[56] Singh, J. et al. TEM, FTIR and XRD analysis of biosynthesized AgNPs. *Mater. Sci. Semicond. Process.* **153**, 107143 (2023).

[57] El Hadrami, A. et al. Chitosan and its effects on plant disease resistance. *Plant Sci.* **179**, 123–129 (2010).

[58] Badawy, M. E. I. & Rabea, E. I. Chitosan is an antimicrobial and antifungal agent. *Carbohydr. Polym.* **137**, 349–359 (2016).

[59] Hilton, S. et al. Synergistic effects of polymers and bioagents in plant disease suppression. *Biol. Control* **130**, 234–242 (2019).

[60] Abdelbaset, E. T. et al. Stimulating banana tree resistance to banana streak virus (BSV) disease by chitosan nanoparticles. *Eur. J. Plant Pathol.*10.1007/s10658-024-02872-7 (2024).

[61] Anderson, J. M. Oxidative stress and antioxidant mechanisms in plants. *Annu. Rev. Plant Physiol.* **36**, 41–57 (1985).

[62] Abd El Rahim, S. Z. & Amein, J. M. Effects of Sclerotium cepivorum on onion physiology. *J. Plant Nutr.* **42**, 891–900 (2019).

[63] Clarkson, J. P. et al. Fungal infection and reductions in soluble sugars in onion. *Physiol. Mol. Plant Pathol.* **60**, 31–42 (2002).

[64] Chang, C. C. et al. Phenolic and flavonoid contents in plant defense. *J. Agric. Food Chem.* **50**, 1128–1132 (2002).

[65] Crowe, M. A. Integrated treatments against soil-borne pathogens. *J. Plant Pathol.* **90**, 37–45 (2008).

[66] Harman, G. E. et al. Trichoderma-induced systemic resistance and antioxidant enzymes in plants. *Front. Plant Sci.* **12**, 676 (2021).

[67] El Saadony, M. T. et al. Antioxidant modulation by chitosan and AgNPs in plant defense responses. *Plant Physiol. Biochem.* **170**, 30–45 (2022).

[68] Badawy, M. E. I. & Rabea, E. I. Antioxidant responses in onion under pathogen stress following bioformulation treatment. *Crop Protect.* **85**, 130–138 (2016).

[69] Hilton, S. et al. Antioxidant enzyme regulation under combined bioagent treatments in plants. *Plant Sci.* **284**, 18–28 (2019).

[70] Mohamed, N. G. et al. Silver nanoparticles–chitosan mixture upregulates xylanase inhibitor (Xip-I) of *Triticum aestivum* infected with *Puccinia graminis*. *Rom. Agric. Res.*10.59665/rar4303 (2026).

[71] Mahmoud, E. Y. et al. Reduction of peanut pod rots and aflatoxin contamination using selected micronutrient nanoparticles. *Not. Bot. Horti Agrobot. Cluj-Napoca* **53**, 14717. 10.15835/nbha53414717 (2025).

